# Patient preferences and willingness-to-pay for therapy in generalized myasthenia gravis: a large-scale discrete choice experiment in China

**DOI:** 10.3389/fimmu.2026.1795988

**Published:** 2026-05-21

**Authors:** Yafang Xu, Ling Feng, Mingjie Zhang, Jing Zhou, Hongwei Xiao, Yuwen Ni, Rui Wang, Yueyue He, Qian Jiang, Jianyu Peng, Hong Chen, Hongyu Zhou, Dingxian He, Sushan Luo, Jianying Xi, Jie Song, Chong Yan, Jiahong Lu, Chongbo Zhao

**Affiliations:** 1 Huashan Rare Disease Center and Department of Nursing, Huashan Hospital, National Center for Neurological Disorders, Fudan University, Shanghai, China; 2 Department of Neurology, West China Hospital, Sichuan University/West China School of Nursing, Sichuan University, Chengdu, China; 3 Huashan Rare Disease Center and Department of Neurology, Huashan Hospital, National Center for Neurological Disorders, Fudan University, Shanghai, China

**Keywords:** discrete choice experiment, Myasthenia Gravis, patient preferences, therapy, willingness to pay

## Abstract

**Background:**

Generalized myasthenia gravis (gMG) is a rare, chronic autoimmune disorder that imposes a substantial disease burden in China. As novel therapeutic options emerge, understanding patient preferences has become essential for treatment decision-making. This study aimed to assess treatment preferences and willingness to pay (WTP) among Chinese gMG patients.

**Methods:**

This multicenter, cross-sectional survey was conducted from March to August 2025 among patients with gMG in China. A discrete choice experiment was used to quantify patient preferences by presenting hypothetical treatment scenarios defined by eight key attributes. A mixed Logit regression model was used to identify preference drivers, and WTP was estimated to reflect the monetary value assigned to changes in treatment attributes.

**Results:**

Among the 909 analyzed patients, the mean age was 48.9 ± 13.27 years, and 61.8% were female. Within the selected attribute sets, safety was a key determinant of patient preferences, with consistently favored for lower risks of adverse drug reactions (ADRs) on metabolic diseases, infection, myelosuppression, and liver and kidney function impairment; WTP increased as risk levels decreased. Patients preferred oral administration, followed by subcutaneous injection and intravenous infusion. Infrequent dosing, once every-6-month, weekly or daily, was preferred over 2–3 times daily. Faster onset time of action (≤2 weeks) was favored, and higher out-of-pocket costs were disfavored. Subgroup analyses revealed young patients valued once weekly dosing, subcutaneous injection, and low risk of liver and kidney function impairment than old patients. Patients with high disease burden showed similar preferences for low and moderate ADRs risk. High-income patients favored low ADRs risk, infrequent dosing, subcutaneous injection, and were less sensitive to cost than to low- and medium-income patients.

**Conclusion:**

This large-scale study was the first to investigated treatment preferences and WTP among Chinese patients with gMG. Within the selected attribute sets, patients prioritized safety, infrequent and convenient administration route, affordability, and rapid onset of action. Patients were willing to invest financially in therapies that align with these preferences. Incorporating patient preferences in clinical decision-making and reimbursement policy may improve adherence, reduce disease burden, and enhance quality of life for individuals with gMG in China.

## 1 Introduction

Myasthenia gravis (MG) is a rare autoimmune disorder affecting neuromuscular transmission, typically manifesting as ocular or generalized MG (gMG) (1, 2). The global prevalence of MG is estimated at 10–20 cases per 100,000 population and has risen with advancements in diagnostic techniques, an aging population, and improved survival (3–5). In China, the age and sex adjusted incidence was 0.68 per 100,000 person-years (6), and over 10,000 new cases were reported annually, underscoring a substantial and growing disease burden (6).

Therapeutic decision-making for MG has become increasingly complex. Conventional therapies often fail to achieve satisfactory clinical improvement or sustained disease stability in 30–50% of patients and are associated with notable side effects (7). Meanwhile, newer targeted therapies, such as complement inhibitors, neonatal Fc receptor (FcRn) antagonists, and anti-CD19 monoclonal antibodies, differ in efficacy, safety, route and frequency of administration, and cost, creating challenging trade-offs for patients and clinicians (3).

As this treatment landscape expands, the clinical and policy value of patient preference evidence has grown. Reflecting this paradigm shift, China’s Center for Drug Evaluation issued guidance in 2023 encouraging the integration of patient preference evidence into benefit-risk assessment, regulatory review, market access, and reimbursement deliberations, signaling increasing attention from both regulators and clinicians to the patient voice (8, 9). The discrete choice experiment (DCE) is widely used to quantify patient preferences by presenting hypothetical treatment scenarios that vary across key attributes (10). By choosing between these alternatives, patients reveal the relative importance they assign to each attribute, enabling researchers to assess how individuals make trade-offs among different aspects of care (10). Complementing DCE, willingness-to-pay (WTP) analysis translates these preferences into monetary terms, facilitating value-based decision-making.

Despite these developments, robust, policy-relevant preference data for gMG in China remain scarce. Previous studies investigating gMG treatment preferences from the clinician’s perspective have identified intensity of symptom improvement, onset speed, and duration of effect as top priorities (3). Limited studies involving small samples of gMG patients have suggested preference for treatments characterized by rapid onset of action, convenient and infrequent dosing, high efficacy, and minimal adverse drug reactions (ADRs) (11–13). Existing studies are largely from Western settings, clinician-focused, or constrained by small patient samples, leaving uncertainty regarding how Chinese patients weigh efficacy, safety, and convenience when choosing among therapies (3, 11–14). To address this gap, we employed approaches of both DCE and WTP analyses to investigate treatment preferences among Chinese gMG patients. To our knowledge, this is the first multicenter, large-sample study to rigorously assess patient preferences and WTP for gMG therapies in China, addressing a significant evidence gap in both global and Chinese patient-centered research.

## 2 Methods

### 2.1 Study design

This multicenter, cross-sectional survey was conducted between March and August 2025 to assess treatment preferences and WTP among patients with gMG in China (**Supplementary Table 1**). Patients were recruited as inpatients and outpatients from Fudan University-Affiliated Huashan Hospital and West China Hospital of Sichuan University. Formal random sampling was not employed; instead, a convenience sample was employed, consisting primarily of patients who voluntarily completed the questionnaire.

The study adhered to the Declaration of Helsinki and was approved by the Ethics Review Committees of the Fudan University Affiliated Huashan Hospital (No. 2025018) and West China Hospital of Sichuan University (No.2025489). The study was registered in the Chinese Clinical Trial Registry (Chictr.gov) (ChiCTR2500098725). Informed consent was obtained from all participants.

### 2.2 Participants

Eligible patients were aged 18–70 years with a confirmed diagnosis of gMG (Myasthenia Gravis Foundation of America [MGFA] class II-IV), regardless of serostatus (seronegative, anti-Acetylcholine Receptor [AChR] or anti- Muscle-Specific Kinase [MuSK]-positive). All patients had received at least one of the following treatments: plasma exchange, acetylcholinesterase (AChE) inhibitors, glucocorticoids, non-steroidal immunosuppressants, or targeted biologics. Exclusion criteria included current participation in other interventional clinical trials for MG or inability to complete the study questionnaires.

### 2.3 Identified attributes and levels

The study attributes and levels were identified through a systematic literature review, clinical guidelines, expert consultation, and input from patient representatives. All participating experts more than 10 years of experience in the diagnosis and management of MG. A total of eight attributes were included: onset time of action, administration route, treatment frequency, annual out-of-pocket (OOP) costs, and risks of metabolic diseases, infection, myelosuppression, and liver and kidney function impairment (**Table 1**). The combination of these attributes and levels generated 9,720 possible hypothetical treatment scenarios (one attribute with two levels, five with three levels, one with four levels, and one with five levels). To enhance feasibility and minimize respondent burden, a D-optimal experimental design was implemented in SAS version 9.4, generating 24 choice sets. Each set comprised two forced-choice alternatives plus an opt-out option, allowing patients to indicate a preference for neither option in a real-world context. To minimize respondent fatigue, the 24 choice sets were evenly divided into three questionnaire versions. Internal validity was assessed through repeated choice sets to evaluate consistency; inconsistent responses were excluded from the final analysis.

**Table 1:** Summary of attributes and levels.

Attributes	Levels
Onset time of action (Time to improvement of gMG symptoms from treatment initiation)	·≤ 2 weeks
	·> 2 weeks
Administration route	·Oral
	·Subcutaneous injection (injection time 20 min)
	·Intravenous injection (infusion time ≥ 60 min)
Treatment frequency	·Once daily
	·Once weekly
	·Once biweekly
	·Once every-6-month
	·2–3 times daily
Annual out-of-pocket costs (All gMG treatment-related expenses after reimbursement by health insurance, if applicable.)	·¥10,000
	·¥30,000
	·¥50,000
	·¥100,000
Risk of metabolic diseases (Metabolic diseases include weight gain, central obesity, increased blood pressure, elevated blood glucose, etc.)	·Low
	·Moderate
	·High
Risk of Infection (Infection include upper respiratory tract infection, meningococcal infection, urinary tract infection, etc.)	·Low
	·Moderate
	·High
Risk of myelosuppression (Myelosuppression includes anemia, leukopenia, thrombocytopenia, etc.)	·Low
	·Moderate
	·High
Risk of liver and kidney function impairment	·Low
	·Moderate
	·High

gMG, generalized myasthenia gravis.

### 2.4 Survey and measures

A pilot test in 21 patients recruited at Fudan University Affiliated Huashan Hospital assessed the questionnaire’s clarity, acceptability, and ease of use. To enhance data quality, trained staff administered the questionnaires face to face, reducing the risk of misinterpretation. The final questionnaire comprised three sections: (1) demographics, (2) diagnosis, treatment experience, and satisfaction, and (3) explanation of and levels with instructions for the DCE tasks and choice questions (**Supplementary Materials**). An example treatment choice scenario is presented in **Table 2**. The survey was administered in Chinese. The complete DCE questionnaire is available in the **Supplementary Materials**.

**Table 2:** An example of a choice task in the discrete choice experiment questionnaire.

Which option would you choose? (Please select only one option and mark it with “√”)		
Attribute/Level	Drug A	Drug B
Onset time of action	≤ 2 weeks	> 2 weeks
Administration route	Oral	Intravenous infusion (infusion time ≥ 60 min)
Treatment frequency	Once daily	Once weekly
Risk of metabolic diseases	Low	Moderate
Risk of infection	Moderate	High
Risk of myelosuppression	Low	Moderate
Risk of liver and kidney function impairment	High	Low
Annual out-of-pocket costs	¥30,000	¥50,000
Which do you prefer?	□	□
In reality, would you be willing to choose the treatment option you selected above?	□Yes □No	

A randomized allocation schedule for the three questionnaire versions (A, B, and C) was generated using block randomization in SAS by an independent statistician blinded to study procedures. Patients were assigned in a 1:1:1 ratio to versions A, B, or C. Prior to survey administration, patients received detailed information about the study’s purpose and questionnaire procedures. Patients were informed that choices among hypothetical treatment profiles reflected personal preference and that there were no right or wrong answers.

### 2.5 Statistical analysis

Descriptive statistics were used to summarize the patients’ demographic and clinical characteristics. For continuous data, sample size, mean, standard deviation, median, interquartile range (IQR), minimum, and maximum values were reported. Categorical data were described as frequencies and percentages, excluding missing data from denominators. Treatment satisfaction was scored using a 10-point scale ranging from 1 (extremely dissatisfied) to 10 (very satisfied).

A mixed Logit regression model was applied to analyze patient choice behavior across multiple treatment attributes. Model coefficients were estimated via maximum likelihood estimation. Coefficient magnitude and sign indicated the relative importance and preference for each attribute level. Positive coefficients indicated preference, whereas negative coefficients indicated aversion to corresponding attribute. WTP was calculated by dividing the coefficient of a non-economic attribute by that of the economic attribute (annual OOP costs), reflecting the monetary value patients assigned to changes in treatment attributes. The relative importance of each attribute was subsequently calculated. To quantify the effect of altering each attribute, a baseline treatment scenario was defined (annual OOP costs ¥10,000, onset time >2 weeks, intravenous administration, dosing 2–3 times per day, and high risk for metabolic diseases, infection, myelosuppression, and liver and kidney function impairment). Selection probabilities were calculated by varying one attribute at a time while holding others constant. Subgroup analyses were performed by age (18–50 years vs. > 50 years), MGFA class (II vs. III), Myasthenia Gravis Activities of Daily Living (MG-ADL) score (MG-ADL ≥ 6 vs. MG-ADL ≤ 5), and annual income (< ¥50,000 vs. ¥50,000-100,000 vs. > ¥100,000). The cut-off values for these subgroup categories were chosen according to clinical conventions and evidence from previous studies (15–17). The MGFA IV subgroup was excluded from subgroup analysis because it contained a single patient. All statistical tests were two-sided, with a significance level (α) of 0.05. Confidence intervals (CIs) were reported at the 95% level. All analyses were performed in SAS software, version 9.4.

## 3 Results

### 3.1 Demographic and socioeconomic characteristics

A total of 958 patients met the eligibility criteria and provided informed consent. Five patients were excluded for survey incompletion and 44 for failing validity checks, resulting in 909 patients included in the final analysis.

Demographic characteristics of the study population are summarized in **Table 3**. The mean age was 48.9 ± 13.27 years, and 61.8% (562) were female. Over half of the patients (537, 59.0%) reported having a senior high school education or above. Only 38.5% (350) were employed either full- or part-time, while 61.5% (559) were unemployed, retired, or others. Regarding economic status, 22.6% (205), 24.0% (218) and 26.4% (240) reported an average household annual income below ¥20,000, ¥20,000-50,000, and ¥50,000-100,000, respectively, over the preceding three years. Most patients (883, 97.1%) had health insurance, primarily Urban Employee Basic Medical Insurance (509, 57.6%), whereas commercial health insurance was less common (138, 15.2%).

**Table 3:** The demographic and socioeconomic characteristics of patients with gMG.

Characteristics	Patients (n=909)
Age, years, mean ± SD	48.9 ± 13.27
Sex, n(%)	
Male	347(38.2)
Female	562(61.8)
Highest level of education, n(%)	
Primary school or lower	135(14.9)
Middle school	237(26.1)
High school/vocational secondary school/technical school	199(21.9)
College diploma/college or higher	338(37.1)
Employment, n(%)	
Full-time/part-time job	350(38.5)
Unemployed/retired	411(45.2)
Others	148(16.3)
Household annual income^*^, n(%)	
< ¥20,000	205(22.6)
¥20,000 to 50,000	218(24.0)
¥50,000 to 100,000	240(26.4)
¥100,000 to 300,000	177(19.5)
¥300,000 to 500,000	46(5.1)
>¥500,000	23(2.5)
Health insurance, n(%)	
No	26(2.9)
Yes	883(97.1)
Urban employee basic medical insurance	509(57.6)
Urban and rural resident basic medical insurance	169(19.1)
New rural cooperative medical scheme	205(23.2)
Commercial medical insurance, n(%)	
No	771(84.8)
Yes	138(15.2)

*Household annual income was the average income in the past 3 years, and ¥1= $0.14 in 2025.

gMG, generalized myasthenia gravis; SD, standard deviation.

### 3.2 Disease characteristics and treatment

The median duration of gMG was 30.1 months (IQR:8.62-78.38). Most patients (874, 96.2%) were classified as MGFA II (**Table 4**). Serological testing revealed that 70.4% (640) were antibody-positive, most commonly for anti-AChR antibodies (623, 68.5%). The mean MG-ADL score was 3.0 ± 3.25. More than half of patients (480, 52.8%) reported comorbidities, with the most frequent being hypertension (220, 24.2%) and diabetes (130, 14.3%). Approximately one-third (301, 33.1%) had undergone thymectomy.

**Table 4:** The disease characteristics of patients with gMG.

Characteristics	Patients (n=909)
Duration of gMG^*^, months, median (IQR)	30.1(8.62-78.38)
MGFA class, n(%)	
II	874(96.2)
III	34(3.7)
IV	1(0.1)
Serological testing, n(%)	
Negative	269(29.6)
Positive^#^	640(70.4)
Anti-AChR antibody positive	623(68.5)
Anti-MuSK antibody positive	25(2.8)
Anti-LRP4 antibody positive	3(0.3)
Anti-transient receptor potential calcium channel antibody positive	1(0.1)
MG-ADL total score, mean ± SD	3.0 ± 3.25
Comorbidity, n(%)	
Yes	480(52.8)
No	429(47.2)
Type of comorbidity^#^, n(%)	
Hypertension	220(24.2)
Diabetes	130(14.3)
Hyperlipidemia	61(6.7)
Others	231(25.4)

*Disease duration of gMG was defined as the duration from diagnosis to the date of informed consent.

^#^ One patient may have multiple records within the same variable.

AChR, acetylcholine receptor; gMG, generalized myasthenia gravis; LRP4, low−density lipoprotein receptor−related protein 4; MG-ADL, Myasthenia Gravis-Activities of Daily Living; MGFA, Myasthenia Gravis Foundation of America; MuSK, muscle-specific kinase.

For gMG medication use (**Table 5**), pyridostigmine was most frequently used (856, 94.2%), followed by prednisone (718, 79.0%) and tacrolimus (413, 45.4%). Oral administration was the most common route (906/909, 99.7%), followed by intravenous infusion (356/909, 39.2%), whereas subcutaneous injection was rare (2/909, 0.2%). Most patients (733, 80.7%) reported annual OOP costs under ¥30,000, with 39.1% (355) spending less than ¥10,000 and 41.6% (378) spending between ¥10,000–30,000.

**Table 5:** The treatment information of patients with gMG.

Characteristics	Patients (n=909)
Thymectomy, n(%)	
Yes	301(33.1)
No	607(66.9)
Missing	1
Treatment for gMG^#^	
Pyridostigmine bromide	856(94.2)
Prednisone acetate	718(79.0)
Methylprednisolone	134(14.7)
Azathioprine	112(12.3)
Tacrolimus	413(45.4)
Mycophenolate mofetil	201(22.1)
Efgartigimod	142(15.6)
Immunoglobulin	210(23.1)
Plasmapheresis	65(7.2)
Others	137(15.1)
Treatment modalities^#^	
Oral	906(99.7)
Intravenous infusion	356(39.2)
Subcutaneous injection	2(0.2)
Plasma exchange	65(7.2)
Average annual out-of-pocket costs for gMG treatment^&^	
<¥10000	355(39.1)
¥10,000 to 30,000	378(41.6)
¥30,000 to 50,000	102(11.2)
¥50,000 to 100,000	50(5.5)
>¥100,000	24(2.6)

^#^ One patient may have multiple records within the same variable.

^&^ ¥1= $0.14 in 2025.

**Figure 1:**
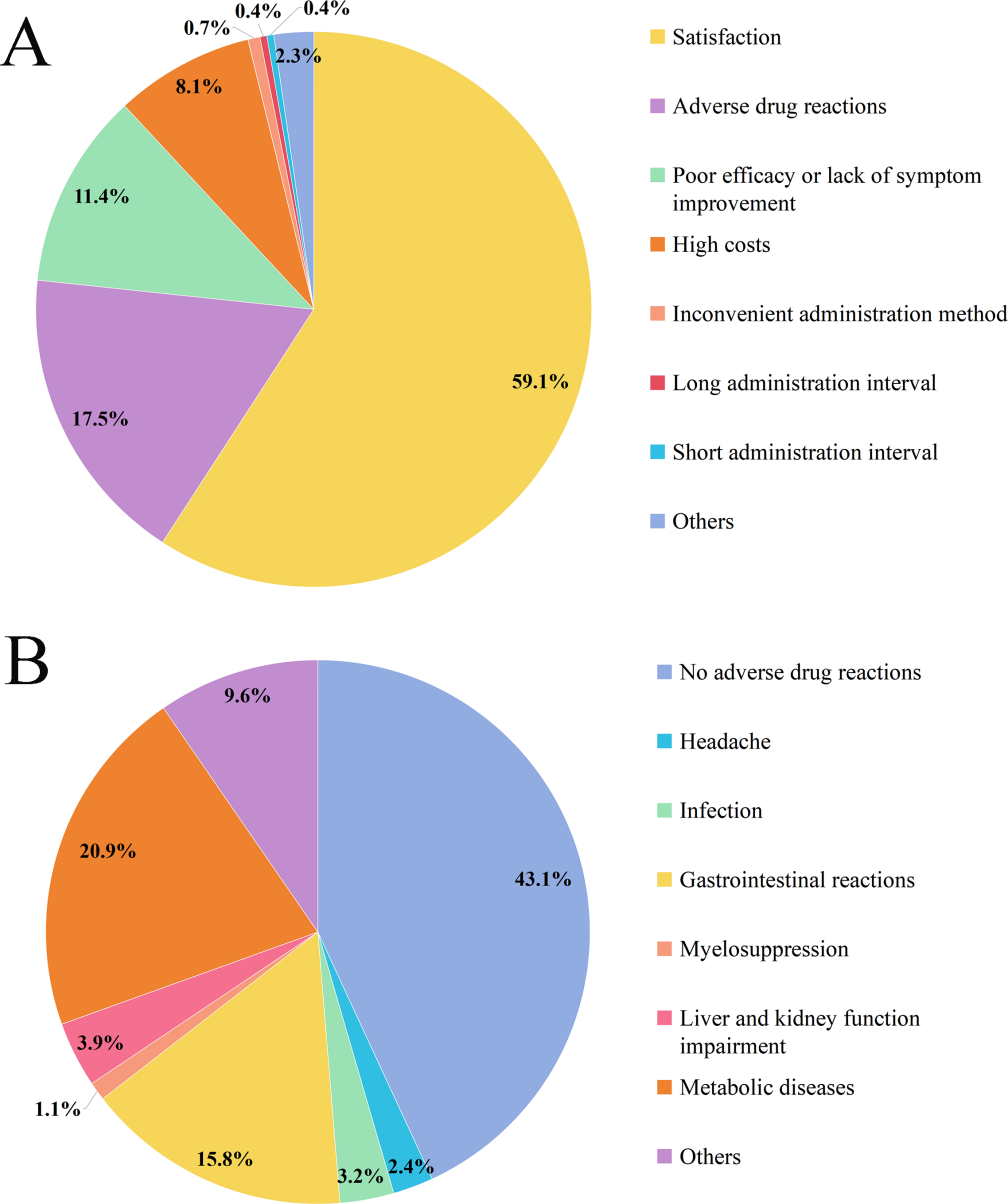
The satisfaction with their current therapies of gMG **(A)** and the most distressing adverse drug reactions **(B)**.

gMG, generalized myasthenia gravis;

On a 10-point scale, the mean treatment satisfaction score was 8.7 ± 1.86. Moreover, 59.1% (537) of patients showed satisfaction with their current therapy (**Figure 1A**). The main reasons for dissatisfaction were ADRs (or side effects, 159, 17.5%), suboptimal efficacy or lack of symptom improvement (104, 11.4%), and high costs (74, 8.1%). Among the types of ADRs that patients found most troublesome, 43.1% (392) reported experiencing none, while metabolic diseases (190, 20.9%) and gastrointestinal reactions (144, 15.8%) were the most frequently cited (**Figure 1B**).

### 3.3 Preferences and willingness to pay of gMG patients

Results from the mixed logit model assessing treatment preferences and WTP are presented in **Table 6**. Statistically significant differences were observed across most levels of all attributes. For treatment frequency, significant differences were found for once daily, once weekly, and once every-6-month dosing, whereas once biweekly was not statistically significant. Compared with an onset time of action > 2 weeks, patients preferred a faster onset (≤2 weeks; preference coefficient: 0.157, 95% CI: 0.105–0.209, *p* < 0.0001), with a corresponding WTP of ¥4,000 per year. Oral administration was the most preferred route (0.241, 95% CI: 0.166–0.316, *p* < 0.0001), followed by subcutaneous injection (0.108, 95% CI: 0.035–0.180, *p* = 0.0035) over intravenous infusion, with WTPs of ¥7,000 and ¥3,000, respectively. Less frequent dosing was favored: once every-6-month (0.396, 95%CI: 0.298–0.494, *p* < 0.0001; WTP ¥11,000), once daily (0.284, 95% CI: 0.176–0.391, *p* < 0.0001; WTP ¥8,000), and once weekly (0.109, 95% CI: 0.008–0.210, *p* = 0.0352; WTP ¥3,000), compared to 2–3 times daily. Dosing every 2 weeks did not significantly affect patient choice. The risks of metabolic diseases, infection, myelosuppression, and liver and kidney function impairment substantially influenced patients’ preferences. Patients strongly preferred treatments with low risk compared with high risk of myelosuppression (0.565, 95% CI: 0.491–0.639, *p* < 0.0001; WTP ¥16,000); moderate risk was also preferred over high (0.405, 95% CI: 0.327–0.483, *p* < 0.0001; WTP ¥11,000). Similar patterns and WTP estimates were observed for risks of metabolic diseases, infection, and liver and kidney function impairment (all *p* < 0.0001). Increasing OOP costs was strongly disfavored (coefficient: -0.363, 95% CI: -0.393–0.334, *p* < 0.0001). The attributes with the highest relative importance were risk of myelosuppression (19.2%), followed by treatment frequency (16.7%), and risk of metabolic diseases (16.7%) (**Supplementary Figure 1**).

**Table 6:** Analysis of patients’ medication preferences and willingness to pay.

Attributes (Levels)	Preference coefficient	95%CI	P value	Willingness-to-pay (per year) ^&^
Onset time of action				
≤2 Weeks	0.157	(0.105-0.209)	<0.0001	¥4,000
>2 Weeks	Ref	--	--	--
Administration route				
Oral	0.241	(0.166-0.316)	<0.0001	¥7,000
Subcutaneous injection (20 min)	0.108	(0.035-0.180)	0.0035	¥3,000
Intravenous infusion (≥60 min)	Ref	--	--	--
Treatment frequency				
Once daily	0.284	(0.176-0.391)	<0.0001	¥8,000
Once weekly	0.109	(0.008-0.210)	0.0352	¥3,000
Once biweekly	0.058	(-0.050-0.166)	0.2944	¥2,000
Once every-6-month	0.396	(0.298-0.494)	<0.0001	¥11,000
2–3 times daily	Ref	--	--	--
Metabolic diseases risk				
Low	0.477	(0.401-0.552)	<0.0001	¥13,000
Moderate	0.366	(0.297-0.436)	<0.0001	¥10,000
High	Ref		--	--
Infection risk				
Low	0.419	(0.344-0.494)	<0.0001	¥12,000
Moderate	0.308	(0.233-0.383)	<0.0001	¥8,000
High	Ref	--	--	--
Myelosuppression risk				
Low	0.565	(0.491-0.639)	<0.0001	¥16,000
Moderate	0.405	(0.327-0.483)	<0.0001	¥11,000
High	Ref	--	--	--
Liver and kidney function impairment				
Low	0.463	(0.383-0.543)	<0.0001	¥13,000
Moderate	0.343	(0.272-0.413)	<0.0001	¥9,000
High	Ref	--	--	--
Annual out-of-pocket costs	-0.363	(-0.393- -0.334)	<0.0001	

^&^ ¥1= $0.14 in 2025.

CI, Confidence interval.

### 3.4 The selection probability of gMG patients

**Figure 2:**
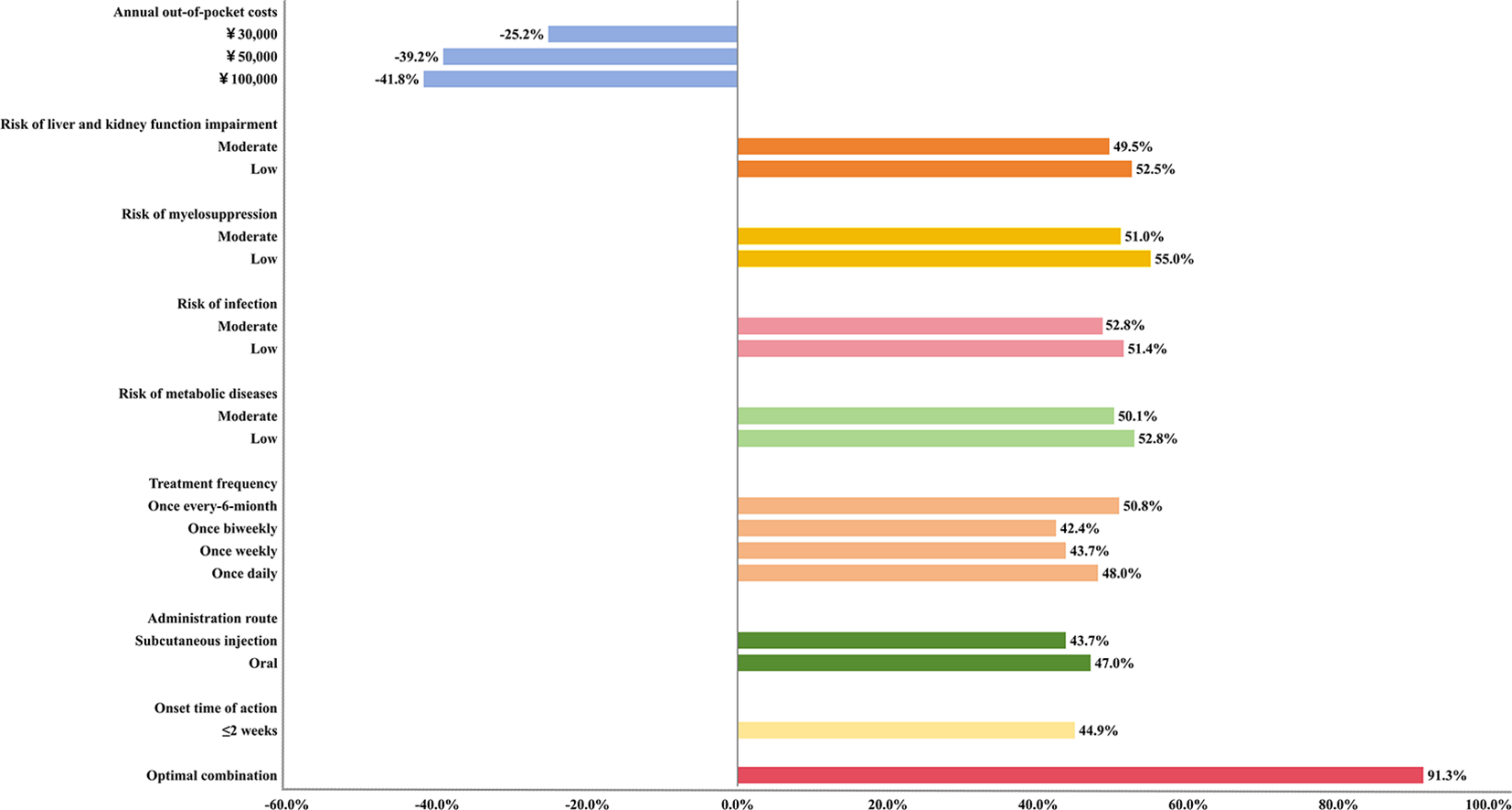
The selection probability of gMG patients. The reference scenario was defined as an annual out-of-pocket cost of ¥10,000, onset time of action >2 weeks, intravenous administration, frequency of 2–3 times daily, and high risks of myelosuppression, infection, metabolic diseases, liver and kidney function impairment. The selection probability of the reference scenario was set to 1.0.

**Figure 2** illustrates the changes in treatment selection probability across attribute levels. The reference scenario was defined as annual OOP cost of ¥10,000, onset time of action >2 weeks, intravenous administration, frequency of 2–3 times daily, and high risks of all ADRs. The selection probability of the reference scenario was set to 1.0. When the annual OOP costs increased to ¥50,000, and ¥100,000, the selection probability reduced 0.392, and 0.418. Conversely, shifting key safety related risks from high to low increased probability by up to 0.550 (myelosuppression), 0.528 (metabolic diseases), 0.514 (infection) and 0.525 (liver and kidney function impairment). Under the optimal scenario (onset time of action ≤2 weeks, oral administration, every-6-month, low risk for all ADRs, and ¥10,000/year cost), predicted selection probability increased by 0.913 relative to baseline.

### 3.5 Subgroup analysis

Subgroup analyses revealed meaningful differences in preferences. Old patients (>50 years, n=469) prioritized once daily dosing (preference coefficient: 0.378 vs. 0.189), and once every-6-month dosing (0.437 vs. 0.364), and greater risk aversion toward myelosuppression (low risk: 0.699 vs. 0.424; moderate risk: 0.466 vs. 0.416) and metabolic diseases (moderate risk: 0.426 vs. 0.306), compared to young patients (18–50 years, n=440, **Supplementary Table 2**, **3**). While young patients (18–50 years) paid more attention to low frequency dosing (preference coefficient: once weekly 0.130 vs. 0.088), subcutaneous injection (0.122 vs. 0.093), and liver and kidney function impairment (0.482 vs. 0.450) than old patients (>50 years). Compared with MGFA class III group (n=34), patients with MGFA class II (n=874) expressed stronger preferences for oral administration (0.255 vs. -0.076), once weekly dosing (0.125 vs. -0.271), low risk of infection disease (0.433 vs. 0.059) and myelosuppression (0.575 vs. 0.352; moderate risk: 0.416 vs. 0.149, **Supplementary Table 4**, **5**). Patients with MGFA class III, however, showed stronger preferences for once-daily dosing (0.832 vs 0.265) than the MGFA class II group. Treatment preferences were similar between patients with high disease burden (MG-ADL≥6, n=182) and low burden (MG-ADL ≤ 5, n=727, **Supplementary Table 6**, **7**). Notably, in the high-burden group, preference coefficients for low and moderate risks were similar across risk domains. High-income patients (>¥100,000, n=246) favored low risks of metabolic diseases, infection, myelosuppression, and liver and kidney function impairment, preferred infrequent (once weekly, once biweekly, or once every-6-month), subcutaneous injection, and were less sensitive to cost than low- and medium-income groups (**Supplementary Tables 8**-**10**). Across all subgroups, observed trends in WTP and preference coefficients were consistent.

## 4 Discussion

This multicenter study represents, to our knowledge, the first effort to systematically quantify treatment preferences among Chinese patients with gMG, thereby addressing key gaps in understanding patient-centered needs. The findings provide actionable guidance for clinical practice and health policy optimization in China. Among the 909 analyzed patients, all included attributes significantly influenced preferences and the WTP magnitude closely mirrored the pattern of preference coefficients. Patients favored therapies that minimized risks of ADRs, and valued the convenience of oral and infrequent (once every-6-month) dosing, rapid onset (≤2 weeks), and low OOP costs. Notably, the risk of myelosuppression, treatment frequency, and the risk of metabolic diseases emerged as the most influential considerations. Subgroup analyses further revealed that patient age, MGFA class, disease burden (MG-ADL score), and income contributed to heterogeneity in patient preferences.

The demographic and socioeconomic characteristics of patients with gMG in this study were consistent with prior nationwide Chinese surveys (6, 15, 18). The annual household income of gMG patients was lower than the national average (19), which was likely attributable to a low employment rate (38.5%) within this population. This pattern is consistent with a Japanese study, in which 31.3% of patients reported unemployment or an involuntary job transfer following the onset of MG. Furthermore, 35.9% of patients experienced a reduction in income, and 47.1% had reductions of ≥50% of their previous total income (20). Notably, we reported higher proportions of MGFA class II (96.2%) patients than earlier studies (15, 21–23). This may be attributed to the presence of selection bias, and the hospital-based recruitment may overrepresent maintenance outpatient population, most of whom were stable at MGFA class II with standard care. For these mildly affected patients, the clinical challenge has shifted from disease control to improve quality of life, such as reducing ADRs severity and the impact of treatment on daily living. The introduction of safer, more convenient therapies may therefore help address the limitations of conventional regimens and improve treatment adherence. Given the 97.1% of patients covered by health insurance, the majority reported annual OOP costs below ¥30,000. This pattern is consistent with previous economic analyses among Chinese MG patients (24).

Within the selected attribute set, patients with gMG placed high value on lower treatment-related risks. Patients showed the highest preference coefficient (0.565) and WTP (¥16,000) for reducing the risk of myelosuppression, followed by metabolic diseases (0.477; ¥13,000), liver and kidney function impairment (0.463; ¥13,000), and infection (0.419; ¥12,000). Preferences coefficients and WTP values decreased with increasing risk of ADRs, underscoring a strong desire to avoid serious, potentially life-threatening events such as myelosuppression. This emphasis is clinically consistent with conventional regimens’ ADRs burden, with more than half of MG patients reporting such events (25). For example, long-term corticosteroid treatment is associated with increased risks of diabetes mellitus, osteoporosis, hormonal and mood disorders (7). Gastrointestinal reactions are very common and intolerable in some patients receiving pyridostigmine (16). Immunosuppressants are also associated with hematologic toxicity, myelosuppression, infection, and gastrointestinal reactions. The incidences of hepatotoxicity and myelosuppression were 15.2% and 9.1% in MG patients receiving azathioprine (26). Furthermore, in patients with comorbidities, avoiding ADRs is not merely preferable but essential, which helps explain why even moderate risk levels (e.g., moderate myelosuppression risk) still garnered positive WTP (¥11,000). Patient-reported outcome studies indicated that 33%-47% of patients with gMG remain dissatisfied with symptom management by conventional therapies (27, 28), highlighting the need for newer treatment options. Recent therapeutic advances, including neonatal FcRn inhibitors (such as rozanolixizumab, efgartigimod) and complement inhibitors (such as zilucoplan, eculizumab), have demonstrated favorable safety profiles and efficacy in phase 3 trials, addressing major safety concerns while improving symptom control (23, 29).

Convenience, both frequency and route of administration, was also highly valued. The strongest preferences were for dosing once every-6-month (preference coefficient: 0.396, WTP: ¥11,000), followed by once daily (0.284, ¥8,000) and once weekly (0.109, ¥3,000). Given the gMG requiring lifelong maintenance therapy, frequent dosing increased the risk of non-adherence; correspondingly, extended dosing intervals (every-6-month) were most preferred. Daily and weekly dosing schedules may be easier for patients to remember. Interestingly, biweekly dosing was not significantly preferred over 2–3 times daily. This unexpected lack of preference for biweekly dosing is understandable in the context of the Chinese healthcare setting. Unlike daily oral medications that can be administered at home, biweekly dosing for gMG usually involves intravenous infusion and frequent hospital visits. The logistical burden of travel and waiting times likely offsets the convenience of less frequent dosing, imposing a significant hidden burden. Moreover, patients may perceive daily dosing as providing more consistent symptom control, whereas longer intervals could raise concerns about waning drug efficacy before the next administration. Patients were also willing to pay approximately ¥7,000 and ¥3,000 annually to switch from intravenous infusion to oral therapy and subcutaneous injection, respectively. This pattern aligned with prior surveys in Europe and underscored the value patients place on home-based treatment, which significantly reduces interference with their daily routines (30, 31). Supporting this, a phase IIIb study revealed that 76.9% of gMG patients preferred once daily injection with zilucoplan over their previous intravenous regimens (ravulizumab or eculizumab) (32). The main reason was the burden of hospital-based infusions, including travel and long administration times. Additionally, under current treatment, oral administration was the most common route, followed by intravenous infusion, while subcutaneous injection was rare. However, patients expressed a preference for subcutaneous over intravenous administration. This discrepancy can be attributed to the timing of regulatory approvals for subcutaneous therapies in China. Prior to 2025, only one subcutaneous drug, efgartigimod SC, was available for gMG (approved in July 2024), resulting in limited real-world use (33). Near our study period (March–August 2025), two additional agents—rozanolixizumab (approved April 2025) and zilucoplan (approved September 2025)—received regulatory approval (34, 35). Consequently, most patients in our study had not yet accessed subcutaneous regimens, which explains why only two participants actually received subcutaneous injection therapy. Affordability emerged as critical constraint, with rising OOP costs associated with pronounced negative utility (coefficient: −0.363). Increasing annual OOP expenditure from ¥10,000 to ¥50,000 or ¥100,000 reduced preference probability by 0.392 and 0.418. These findings indicate that highly preferred therapies (e.g., low risk, low frequency) may be inaccessible for many patients due to cost. Furthermore, we observed high WTP (¥16,000 for reducing myelosuppression risk) despite low affordability, with 73.0% of patients’ household annual income below ¥100,000. This tension is clear in two new National Reimbursement Drug List (NRDL) -listed targeted therapies. Efgartigimod, priced at ¥5,608 per vial under the negotiated reimbursement, costs approximately ¥44,864 per cycle for patients weighing over 40 kg (36), with continuation decisions contingent on clinical response. After accounting for reimbursement rates ranging from 50% to 80%, patients’ OOP costs still range from ¥8,972 to ¥22,432 per cycle. Similarly, eculizumab is priced at ¥2,518 per vial, leading to an annual treatment cost around ¥251,800, with OOP costs spanning ¥50,360 to ¥125,900 (36). These costs far exceed the annual income of a large share of gMG patients, underscoring a persistent access barrier despite reimbursement. Consistent with our findings, a national survey of Chinese rare disease patients reported that 51.2% were unable to obtain required medicines due to cost, emphasizing the ongoing challenge of drug affordability in rare diseases (37).

Subgroup analyses demonstrated significant variation in treatment preferences. Young patients prioritized convenience (subcutaneous route and once weekly dosing), probably because these regimens minimized interference work and social activities. In addition, young patients were more concerned about the potential impact of treatment on liver and kidney function impairment, likely due to a longer anticipated life span and a desire to avoid long-term complications that could affect future health or quality of life. Old patients demonstrated a strong aversion to myelosuppression and metabolic diseases, regardless of whether the risk was moderate or low. This heightened concern may reflect reduced bone marrow reserve and increased vulnerability to complications, such as infection or bleeding, that can arise from myelosuppression. Patients with MGFA class III, who experience more severe symptoms, placed a higher value on once-daily dosing, perhaps because a regular daily regimen provides a sense of stability and control over disease progression, which was crucial for those with more advanced gMG. In contrast, patients with MGFA class II, who had milder disease, focused more on treatment convenience (such as oral administration and once weekly dosing) and on minimizing risks of infection and myelosuppression. For this subgroup, treatments that did not reduce their quality of life and have fewer ADRs were prioritized. In the high-burden subgroup (MG-ADL≥6), we observed similar preference coefficients for low and moderate risks across different risk attributes, suggesting that they may prioritize any reduction in ADRs risk over pursuing the lowest possible risk level. Therefore, it is essential to tailor medication prescriptions according to the preferences and specific needs of different patients.

In this study, 59.1% of patients reported satisfaction with their current treatment, with a mean score of 8.7 out of 10. However, this contrasted with their stated willingness to pay up to ¥16,000 per year to reduce ADRs. This contradiction likely reflects the long-standing lack of new therapies for gMG patients in China, as FcRn inhibitors have only recently been approved or included in insurance coverage. Therefore, patients’ reported satisfaction may primarily stem from stable disease control with conventional treatments and the absence of better alternatives. While the DCE depicting an ideal therapy scenario revealed patients’ strong preference for improved quality of life and treatments that are safer and more convenient. In practice, these preferences are often constrained by patients’ affordability. Policymakers should therefore place greater weight on safety and convenience when evaluating gMG therapies for coverage. Given affordability constraints, a unified multi-tiered financing system with stronger coverage for long-term outpatient MG treatments is required (38). In tandem, policymakers could raise reimbursement rates and outpatient pooling ceilings to improve financial protection (39). Complementary supply-side measures, such as accelerated approvals, targeted tax credits, fee waivers, and priority review vouchers, could spur rare-disease R&D and enable earlier access to new therapies (39, 40).

This study had several limitations. First, the selection bias may have occurred. Patients were recruited from hospitals and may have been more actively engaged in seeking medical care than the broader gMG population. As a result, the sample only included individuals currently receiving treatment, potentially underrepresenting those less engaged with the healthcare system and possibly missing those with the greatest unmet medication needs. Second, over 90% of the patients were MGFA class II, with few severe (Type III or higher) cases, constraining insights from more severe subgroups. This pronounced imbalance limits the robustness of subgroup analyses, and any conclusions regarding treatment preferences among more severe patients should be interpreted with caution. Third, study sites were limited, as gMG care in China is largely concentrated in tertiary hospitals across regions. We selected patients from two tertiary hospitals each in the eastern and western regions. Although this aimed to cover diverse geographical areas, a significant concentration of patients in certain provinces limited the study’s broader national representativeness. In addition, the age range of study participants was restricted to 18–70 years to ensure cognitive ability to independently complete the DCE survey. As a result, potential bias against older patients exists, as the perspectives of individuals over 70 years may not have been adequately captured.

This large-scale, multicenter study is, to our knowledge, the first to characterize treatment preferences and WTP among Chinese patients with gMG, providing novel insights into patient-centered priorities. Safety, treatment frequency, administration route, cost, and onset time of action were key drivers of treatment choice, and patients were willing to pay for therapies aligned with these preferences. Embedding patient preferences in clinical decision-making and reimbursement policy may improve adherence, and enhance quality of life for individuals with gMG in China. Our findings provide valuable insights that may help clinicians evaluate new treatment options for patients seeking treatment optimization or improved outcomes.

## Data Availability

The raw data supporting the conclusions of this article will be made available by the authors, without undue reservation.
