# Supplementary material for: Patient preferences and willingness-to-pay for therapy in generalized myasthenia gravis: a large-scale discrete choice experiment in China
Source: Front Immunol. 2026 May 21;17:1795988. doi: 10.3389/fimmu.2026.1795988 (PMC13233229; doi:10.3389/fimmu.2026.1795988)
Supplement: Supplementary file 1 [file DataSheet1.docx]

Supplementary Material

# Supplementary Figures and Tables

## Supplementary Figures

**Supplementary Figure 1. Relative importance weights of each attribute**

## Supplementary Tables

**Supplementary Table 1. Distribution of patients' residential locations**

| **Region** | **Number of patients** |
| --- | --- |
| East China | 649 |
| Southwest China | 119 |
| Central China | 31 |
| South China | 10 |
| Northeast China | 14 |
| Northwest China | 18 |
| North China | 7 |
| Missing | 35 |
| Total | 909 |

**Supplementary Table 2. Analysis of patients’ medication preferences and willingness to pay (age 18-50 years)**

| Attributes (Levels) | Preference coefficient | 95%CI | P value | Willingness-to-pay (per year) |
| --- | --- | --- | --- | --- |
| Onset time |  |  |  |  |
| ≤2 Weeks | 0.144 | (0.070~0.219) | 0.0001 | ¥4,000 |
| >2 Weeks | -- |  | -- | -- |
| Administration route |  |  |  |  |
| Oral | 0.251 | (0.145~0.357) | <0.0001 | ¥7,000 |
| Subcutaneous Injection (20 min) | 0.122 | (0.018~0.227) | 0.0220 | ¥4,000 |
| Intravenous infusion (≥60 min) | -- |  | -- | -- |
| Treatment frequency |  |  |  |  |
| Once daily | 0.189 | (0.033~0.345) | 0.0176 | ¥5,000 |
| Weekly | 0.130 | (-0.014~0.275) | 0.0775 | ¥4,000 |
| Biweekly | -0.003 | (-0.155~0.149) | 0.9676 | ¥0 |
| Every-6-month | 0.364 | (0.222~0.506) | <0.0001 | ¥10,000 |
| Daily 2-3 times | -- |  | -- | -- |
| Metabolic disease risk |  |  |  |  |
| Low | 0.452 | (0.345~0.560) | <0.0001 | ¥13,000 |
| Moderate | 0.306 | (0.208~0.404) | <0.0001 | ¥9,000 |
| High | -- |  | -- | -- |
| Infection risk |  |  |  |  |
| Low | 0.401 | (0.295~0.506) | <0.0001 | ¥12,000 |
| Moderate | 0.329 | (0.222~0.435) | <0.0001 | ¥9,000 |
| High | -- |  | -- | -- |
| Myelosuppression risk |  |  |  |  |
| Low | 0.424 | (0.319~0.529) | <0.0001 | ¥12,000 |
| Moderate | 0.339 | (0.230~0.447) | <0.0001 | ¥10,000 |
| High | -- |  | -- | -- |
| Liver and kidney function impairment |  |  |  |  |
| Low | 0.482 | (0.367~0.597) | <0.0001 | ¥14,000 |
| Moderate | 0.349 | (0.250~0.448) | <0.0001 | ¥10,000 |
| High | -- |  | -- | -- |
| Annually out-of-pocket costs | -0.348 | (-0.391~-0.305) | <0.0001 |  |

**Supplementary Table 3. Analysis of patients’ medication preferences and willingness to pay (age >50 years)**

| Attributes (Levels) | Preference coefficient | 95%CI | P value | Willingness-to-pay  (per year) |
| --- | --- | --- | --- | --- |
| Onset time |  |  |  |  |
| ≤2 Weeks | 0.175 | (0.101~0.249) | <0.0001 | ¥5,000 |
| >2 Weeks | -- |  | -- | -- |
| Administration route |  |  |  |  |
| Oral | 0.236 | (0.129~0.343) | <0.0001 | ¥6,000 |
| Subcutaneous Injection (20 min) | 0.093 | (-0.009~0.194) | 0.0747 | ¥2,000 |
| Intravenous infusion (≥60 min) | -- |  | -- | -- |
| Treatment frequency |  |  |  |  |
| Once daily | 0.378 | (0.228~0.529) | <0.0001 | ¥10,000 |
| Weekly | 0.088 | (-0.057~0.232) | 0.2341 | ¥2,000 |
| Biweekly | 0.109 | (-0.047~0.264) | 0.1720 | ¥3,000 |
| Every-6-month | 0.437 | (0.300~0.575) | <0.0001 | ¥12,000 |
| Daily 2-3 times | -- |  | -- | -- |
| Metabolic disease risk |  |  |  |  |
| Low | 0.503 | (0.396~0.610) | <0.0001 | ¥13,000 |
| Moderate | 0.426 | (0.327~0.525) | <0.0001 | ¥11,000 |
| High | -- |  | -- | -- |
| Infection risk |  |  |  |  |
| Low | 0.456 | (0.348~0.563) | <0.0001 | ¥12,000 |
| Moderate | 0.309 | (0.203~0.416) | <0.0001 | ¥8,000 |
| High | -- |  | -- | -- |
| Myelosuppression risk |  |  |  |  |
| Low | 0.699 | (0.594~0.805) | <0.0001 | ¥18,000 |
| Moderate | 0.466 | (0.354~0.578) | <0.0001 | ¥12,000 |
| High | -- |  | -- | -- |
| Liver and kidney function impairment |  |  |  |  |
| Low | 0.450 | (0.336~0.563) | <0.0001 | ¥12,000 |
| Moderate | 0.332 | (0.231~0.434) | <0.0001 | ¥9,000 |
| High | -- |  | -- | -- |
| Annually out-of-pocket costs | -0.379 | (-0.421~-0.336) | <0.0001 |  |

**Supplementary Table 4.** **Analysis of patients’ medication preferences and willingness to pay (MGFA class II)**

| Attributes (Levels) | Preference coefficient | 95%CI | P value | Willingness-to-pay  (per year) |
| --- | --- | --- | --- | --- |
| Onset time |  |  |  |  |
| ≤2 Weeks | 0.163 | (0.110~0.216) | <0.0001 | ¥4,000 |
| >2 Weeks | -- |  | -- | -- |
| Administration route |  |  |  |  |
| Oral | 0.255 | (0.179~0.332) | <0.0001 | ¥7,000 |
| Subcutaneous Injection (20 min) | 0.103 | (0.029~0.177) | 0.0064 | ¥3,000 |
| Intravenous infusion (≥60 min) | -- |  | -- | -- |
| Treatment frequency |  |  |  |  |
| Once daily | 0.265 | (0.155~0.375) | <0.0001 | ¥7,000 |
| Weekly | 0.125 | (0.022~0.229) | 0.0179 | ¥3,000 |
| Biweekly | 0.056 | (-0.054~0.167) | 0.3163 | ¥2,000 |
| Every-6-month | 0.404 | (0.304~0.504) | <0.0001 | ¥11,000 |
| Daily 2-3 times | -- |  | -- | -- |
| Metabolic disease risk |  |  |  |  |
| Low | 0.481 | (0.404~0.558) | <0.0001 | ¥13,000 |
| Moderate | 0.369 | (0.299~0.440) | <0.0001 | ¥10,000 |
| High | -- |  | -- | -- |
| Infection risk |  |  |  |  |
| Low | 0.433 | (0.356~0.509) | <0.0001 | ¥12,000 |
| Moderate | 0.314 | (0.237~0.390) | <0.0001 | ¥9,000 |
| High | -- |  | -- | -- |
| Myelosuppression risk |  |  |  |  |
| Low | 0.575 | (0.499~0.651) | <0.0001 | ¥16,000 |
| Moderate | 0.416 | (0.336~0.495) | <0.0001 | ¥11,000 |
| High | -- |  | -- | -- |
| Liver and kidney function impairment |  |  |  |  |
| Low | 0.465 | (0.383~0.547) | <0.0001 | ¥13,000 |
| Moderate | 0.346 | (0.274~0.418) | <0.0001 | ¥9,000 |
| High | -- |  | -- | -- |
| Annually out-of-pocket costs | -0.366 | (-0.396~-0.335) | <0.0001 |  |

**Supplementary Table 5.** **Analysis of patients’ medication preferences and willingness to pay (MGFA class III)**

| Attributes (Levels) | Preference coefficient | 95%CI | P value | Willingness-to-pay  (per year) |
| --- | --- | --- | --- | --- |
| Onset time |  |  |  |  |
| ≤2 Weeks | 0.022 | (-0.261~0.305) | 0.8789 | ¥1,000 |
| >2 Weeks | -- |  | -- | -- |
| Administration route |  |  |  |  |
| Oral | -0.076 | (-0.467~0.316) | 0.7053 | -¥2,000 |
| Subcutaneous Injection (20 min) | 0.202 | (-0.172~0.576) | 0.2891 | ¥5,000 |
| Intravenous infusion (≥60 min) | -- |  | -- | -- |
| Treatment frequency |  |  |  |  |
| Once daily | 0.832 | (0.246~1.418) | 0.0054 | ¥22,000 |
| Weekly | -0.271 | (-0.816~0.274) | 0.3290 | -¥7,000 |
| Biweekly | 0.132 | (-0.439~0.703) | 0.6507 | ¥3,000 |
| Every-6-month | 0.260 | (-0.244~0.763) | 0.3128 | ¥7,000 |
| Daily 2-3 times | -- |  | -- | -- |
| Metabolic disease risk |  |  |  |  |
| Low | 0.470 | (0.057~0.883) | 0.0259 | ¥12,000 |
| Moderate | 0.300 | (-0.072~0.673) | 0.1142 | ¥7,000 |
| High | -- |  | -- | -- |
| Infection risk |  |  |  |  |
| Low | 0.059 | (-0.342~0.459) | 0.7736 | ¥2,000 |
| Moderate | 0.246 | (-0.145~0.637) | 0.2172 | ¥6,000 |
| High | -- |  | -- | -- |
| Myelosuppression risk |  |  |  |  |
| Low | 0.352 | (-0.032~0.736) | 0.0721 | ¥9,000 |
| Moderate | 0.149 | (-0.268~0.566) | 0.4836 | ¥4,000 |
| High | -- |  | -- | -- |
| Liver and kidney function impairment |  |  |  |  |
| Low | 0.541 | (0.118~0.963) | 0.0122 | ¥14,000 |
| Moderate | 0.418 | (0.031~0.806) | 0.0345 | ¥11,000 |
| High | -- |  | -- | -- |
| Annually out-of-pocket costs | -0.386 | (-0.539~-0.233) | <0.0001 |  |

**Supplementary Table 6.** **Analysis of patients’ medication preferences and willingness to pay (MG-ADL≥6)**

| Attributes (Levels) | Preference coefficient | 95%CI | P value | Willingness-to-pay  (per year) |
| --- | --- | --- | --- | --- |
| Onset time |  |  |  |  |
| ≤2 Weeks | 0.162 | (0.046~0.279) | 0.0063 | ¥4,000 |
| >2 Weeks | -- |  | -- | -- |
| Administration route |  |  |  |  |
| Oral | 0.176 | (0.009~0.344) | 0.0394 | ¥5,000 |
| Subcutaneous Injection (20 min) | 0.124 | (-0.037~0.286) | 0.1314 | ¥3,000 |
| Intravenous infusion (≥60 min) | -- |  | -- | -- |
| Treatment frequency |  |  |  |  |
| Once daily | 0.322 | (0.083~0.561) | 0.0084 | ¥8,000 |
| Weekly | 0.040 | (-0.190~0.270) | 0.7313 | ¥1,000 |
| Biweekly | -0.013 | (-0.252~0.226) | 0.9160 | ¥0 |
| Every-6-month | 0.276 | (0.067~0.486) | 0.0097 | ¥7,000 |
| Daily 2-3 times | -- |  | -- | -- |
| Metabolic disease risk |  |  |  |  |
| Low | 0.455 | (0.284~0.627) | <0.0001 | ¥12,000 |
| Moderate | 0.425 | (0.269~0.582) | <0.0001 | ¥11,000 |
| High | -- |  | -- | -- |
| Infection risk |  |  |  |  |
| Low | 0.387 | (0.216~0.558) | <0.0001 | ¥10,000 |
| Moderate | 0.405 | (0.236~0.574) | <0.0001 | ¥10,000 |
| High | -- |  | -- | -- |
| Myelosuppression risk |  |  |  |  |
| Low | 0.490 | (0.326~0.653) | <0.0001 | ¥13,000 |
| Moderate | 0.484 | (0.305~0.663) | <0.0001 | ¥13,000 |
| High | -- |  | -- | -- |
| Liver and kidney function impairment |  |  |  |  |
| Low | 0.387 | (0.208~0.566) | <0.0001 | ¥10,000 |
| Moderate | 0.292 | (0.131~0.454) | 0.0004 | ¥8,000 |
| High | -- |  | -- | -- |
| Annually out-of-pocket costs | -0.386 | (-0.453~-0.318) | <0.0001 |  |

**Supplementary Table 7.** **Analysis of patients’ medication preferences and willingness to pay (MG-ADL≤5)**

| Attributes (Levels) | Preference coefficient | 95%CI | P value | Willingness-to-pay  (per year) |
| --- | --- | --- | --- | --- |
| Onset time |  |  |  |  |
| ≤2 Weeks | 0.157 | (0.099~0.216) | <0.0001 | ¥4,000 |
| >2 Weeks | -- |  | -- | -- |
| Administration route |  |  |  |  |
| Oral | 0.253 | (0.169~0.337) | <0.0001 | ¥7,000 |
| Subcutaneous Injection (20 min) | 0.101 | (0.020~0.183) | 0.0144 | ¥3,000 |
| Intravenous infusion (≥60 min) | -- |  | -- | -- |
| Treatment frequency |  |  |  |  |
| Once daily | 0.273 | (0.152~0.394) | <0.0001 | ¥8,000 |
| Weekly | 0.125 | (0.012~0.239) | 0.0304 | ¥3,000 |
| Biweekly | 0.081 | (-0.041~0.203) | 0.1926 | ¥2,000 |
| Every-6-month | 0.429 | (0.317~0.540) | <0.0001 | ¥12,000 |
| Daily 2-3 times | -- |  | -- | -- |
| Metabolic disease risk |  |  |  |  |
| Low | 0.485 | (0.401~0.569) | <0.0001 | ¥13,000 |
| Moderate | 0.350 | (0.272~0.428) | <0.0001 | ¥10,000 |
| High | -- |  | -- | -- |
| Infection risk |  |  |  |  |
| Low | 0.425 | (0.342~0.509) | <0.0001 | ¥12,000 |
| Moderate | 0.285 | (0.201~0.369) | <0.0001 | ¥8,000 |
| High | -- |  | -- | -- |
| Myelosuppression risk |  |  |  |  |
| Low | 0.592 | (0.509~0.676) | <0.0001 | ¥16,000 |
| Moderate | 0.394 | (0.308~0.480) | <0.0001 | ¥11,000 |
| High | -- |  | -- | -- |
| Liver and kidney function impairment |  |  |  |  |
| Low | 0.485 | (0.395~0.575) | <0.0001 | ¥14,000 |
| Moderate | 0.354 | (0.275~0.433) | <0.0001 | ¥10,000 |
| High | -- |  | -- | -- |
| Annually out-of-pocket costs | -0.359 | (-0.393~-0.326) | <0.0001 |  |

**Supplementary Table 8.** **Analysis of patients’ medication preferences and willingness to pay (income<¥50,000 per year)**

| Attributes (Levels) | Preference coefficient | 95%CI | P value | Willingness-to-pay  (per year) |
| --- | --- | --- | --- | --- |
| Onset time |  |  |  |  |
| ≤2 Weeks | 0.195 | (0.118~0.271) | <0.0001 | ¥5,000 |
| >2 Weeks | -- |  | -- | -- |
| Administration route |  |  |  |  |
| Oral | 0.173 | (0.062~0.284) | 0.0023 | ¥4,000 |
| Subcutaneous Injection (20 min) | 0.033 | (-0.071~0.137) | 0.5341 | ¥1,000 |
| Intravenous infusion (≥60 min) | -- |  | -- | -- |
| Treatment frequency |  |  |  |  |
| Once daily | 0.271 | (0.114~0.427) | 0.0007 | ¥7,000 |
| Weekly | -0.019 | (-0.167~0.129) | 0.8020 | ¥0 |
| Biweekly | -0.108 | (-0.267~0.050) | 0.1789 | -¥3,000 |
| Every-6-month | 0.203 | (0.060~0.346) | 0.0054 | ¥5,000 |
| Daily 2-3 times | -- |  | -- | -- |
| Metabolic disease risk |  |  |  |  |
| Low | 0.375 | (0.265~0.486) | <0.0001 | ¥9,000 |
| Moderate | 0.285 | (0.182~0.387) | <0.0001 | ¥7,000 |
| High | -- |  | -- | -- |
| Infection risk |  |  |  |  |
| Low | 0.402 | (0.292~0.512) | <0.0001 | ¥10,000 |
| Moderate | 0.316 | (0.206~0.425) | <0.0001 | ¥8,000 |
| High | -- |  | -- | -- |
| Myelosuppression risk |  |  |  |  |
| Low | 0.509 | (0.402~0.617) | <0.0001 | ¥13,000 |
| Moderate | 0.357 | (0.241~0.472) | <0.0001 | ¥9,000 |
| High | -- |  | -- | -- |
| Liver and kidney function impairment |  |  |  |  |
| Low | 0.449 | (0.331~0.566) | <0.0001 | ¥11,000 |
| Moderate | 0.294 | (0.191~0.398) | <0.0001 | ¥7,000 |
| High | -- |  | -- | -- |
| Annually out-of-pocket costs | -0.407 | (-0.451~-0.362) | <0.0001 |  |

**Supplementary Table 9.** **Analysis of patients’ medication preferences and willingness to pay (income ¥50,000-100,000 per year)**

| Attributes (Levels) | Preference coefficient | 95%CI | P value | Willingness-to-pay  (per year) |
| --- | --- | --- | --- | --- |
| Onset time |  |  |  |  |
| ≤2 Weeks | 0.112 | (0.010~0.214) | 0.0314 | ¥3,000 |
| >2 Weeks | -- |  | -- | -- |
| Administration route |  |  |  |  |
| Oral | 0.255 | (0.109~0.401) | 0.0006 | ¥7,000 |
| Subcutaneous Injection (20 min) | 0.118 | (-0.025~0.261) | 0.1053 | ¥3,000 |
| Intravenous infusion (≥60 min) | -- |  | -- | -- |
| Treatment frequency |  |  |  |  |
| Once daily | 0.390 | (0.175~0.605) | 0.0004 | ¥11,000 |
| Weekly | 0.155 | (-0.044~0.355) | 0.1274 | ¥4,000 |
| Biweekly | 0.137 | (-0.073~0.348) | 0.2016 | ¥4,000 |
| Every-6-month | 0.428 | (0.239~0.618) | <0.0001 | ¥12,000 |
| Daily 2-3 times | -- |  | -- | -- |
| Metabolic disease risk |  |  |  |  |
| Low | 0.561 | (0.410~0.711) | <0.0001 | ¥15,000 |
| Moderate | 0.383 | (0.248~0.517) | <0.0001 | ¥10,000 |
| High | -- |  | -- | -- |
| Infection risk |  |  |  |  |
| Low | 0.365 | (0.218~0.512) | <0.0001 | ¥10,000 |
| Moderate | 0.268 | (0.123~0.413) | 0.0003 | ¥7,000 |
| High | -- |  | -- | -- |
| Myelosuppression risk |  |  |  |  |
| Low | 0.566 | (0.421~0.711) | <0.0001 | ¥15,000 |
| Moderate | 0.370 | (0.220~0.521) | <0.0001 | ¥10,000 |
| High | -- |  | -- | -- |
| Liver and kidney function impairment |  |  |  |  |
| Low | 0.490 | (0.334~0.646) | <0.0001 | ¥13,000 |
| Moderate | 0.380 | (0.241~0.519) | <0.0001 | ¥10,000 |
| High | -- |  | -- | -- |
| Annually out-of-pocket costs | -0.371 | (-0.429~-0.313) | <0.0001 |  |

**Supplementary Table 10. Analysis of patients’ medication preferences and willingness to pay (income >¥100,000 per year)**

| Attributes (Levels) | Preference coefficient | 95%CI | P value | Willingness-to-pay  (per year) |
| --- | --- | --- | --- | --- |
| Onset time |  |  |  |  |
| ≤2 Weeks | 0.143 | (0.041~0.245) | 0.0061 | ¥5,000 |
| >2 Weeks | -- |  | -- | -- |
| Administration route |  |  |  |  |
| Oral | 0.351 | (0.205~0.497) | <0.0001 | ¥12,000 |
| Subcutaneous Injection (20 min) | 0.227 | (0.081~0.372) | 0.0023 | ¥8,000 |
| Intravenous infusion (≥60 min) | -- |  | -- | -- |
| Treatment frequency |  |  |  |  |
| Once daily | 0.232 | (0.020~0.444) | 0.0319 | ¥8,000 |
| Weekly | 0.283 | (0.082~0.483) | 0.0058 | ¥10,000 |
| Biweekly | 0.231 | (0.015~0.447) | 0.0361 | ¥8,000 |
| Every-6-month | 0.720 | (0.522~0.919) | <0.0001 | ¥25,000 |
| Daily 2-3 times | -- |  | -- | -- |
| Metabolic disease risk |  |  |  |  |
| Low | 0.598 | (0.452~0.744) | <0.0001 | ¥21,000 |
| Moderate | 0.496 | (0.359~0.633) | <0.0001 | ¥17,000 |
| High | -- |  | -- | -- |
| Infection risk |  |  |  |  |
| Low | 0.507 | (0.361~0.652) | <0.0001 | ¥17,000 |
| Moderate | 0.353 | (0.204~0.501) | <0.0001 | ¥12,000 |
| High | -- |  | -- | -- |
| Myelosuppression risk |  |  |  |  |
| Low | 0.689 | (0.539~0.839) | <0.0001 | ¥24,000 |
| Moderate | 0.533 | (0.382~0.683) | <0.0001 | ¥18,000 |
| High | -- |  | -- | -- |
| Liver and kidney function impairment |  |  |  |  |
| Low | 0.494 | (0.335~0.652) | <0.0001 | ¥17,000 |
| Moderate | 0.421 | (0.282~0.560) | <0.0001 | ¥14,000 |
| High | -- |  | -- | -- |
| Annually out-of-pocket costs | -0.291 | (-0.349~-0.232) | <0.0001 |  |

# Supplementary Material

**Questionnaire on Treatment Preferences for Generalized Myasthenia Gravis Patients in China**

Dear Participant,

Thank you very much for taking the time to participate in this survey. We aim to understand your perspectives, preferences, and satisfaction regarding drugs for generalized myasthenia gravis (gMG). We would be grateful if you could carefully read the following information and complete the questionnaire.

This questionnaire is distributed to all participants in this study. Participation is entirely voluntary, and all information you provide will be kept confidential. To facilitate your understanding, we have included detailed instructions for completing the questionnaire.

Thank you again for your participation!

**Part One: Personal Basic Information**

Instructions for Completion (Please read carefully before you start filling in!):

1. Please answer all questions carefully. Please fill in with a black rollerball pen, ballpoint pen, or fountain pen. If you make a mistake, draw a single line through the incorrect entry, correct it in the adjacent blank space, and sign with your initials along with the date of correction (e.g., "20.06 LZH 21.03.10". [Do not use correction fluid, erasers, or scribble over mistakes]).
2. Fields marked with an asterisk (*) are optional and can be skipped at your discretion.
3. For multiple-choice items, please mark the box “□” with a "√" to indicate your choice, like☑.
4. The screening number of participant and random number will be provided to you by the researchers.

| **Screening number of participant:** | |
| --- | --- |
| **Date of Birth:** | |
| **Random number:** | |
| **Gender:** □ Male □ Female | **Ethnicity*：**□ Han □ Others |
| **Marital Status*：**□ Married □ Single □ Divorced | |
| **Education Level：**Primary or below □ Middle school □ High school/Vocational secondary school/Technical school □ College diploma □ Bachelor □ Master □ Doctorate | |
| **Employment Status*：**Full-time □ Part-time □ Student □ Unemployed □ Disabled □ Retired □ Other | |
| **Household Income (Average annual income over past 3 years):**  □ <¥20,000 □ ¥20,000–50,000 □ ¥50,000–100,000 □ ¥100,000–300,000 □ ¥300,000–500,000 □ >¥500,000 | |
| **Health Insurance:** □ Yes □ No | |
| **Type of Health Insurance (if applicable):** □ Urban employee basic medical insurance  □ Urban and rural resident basic medical insurance □ New rural cooperative medical scheme | |
| **Commercial Medical Insurance*:** □ Yes □ No | |
| **Type of Commercial Insurance (if applicable)*：**□ Major Medical □ Critical illness □ Others | |
| **Place of Residence:** | |
| **Date of Questionnaire First Completion:** | |

**Part Two: Disease and Treatment Information & Patient Satisfaction of Participants**

Instructions for Completion (Please read carefully before you start filling in!) :

1. Please answer all questions carefully. Please fill in with a black rollerball pen, ballpoint pen, or fountain pen. If you make a mistake, draw a single line through the incorrect entry, correct it in the adjacent blank space, and sign with your initials along with the date of correction (e.g., "20.06 LZH 21.03.10". [Do not use correction fluid, erasers, or scribble over mistakes]).
2. This part of the questionnaire aims to understand your current disease characteristics, medication status, and satisfaction with the current treatment. If you have questions or are unsure about the detailed information regarding your disease or medication, you may refer to your medical records, laboratory test reports, or consult your researcher (attending doctor or nurse) in a timely manner.
3. All selective items, please mark the box “□” with a "√" to indicate your choice, like☑.

| **一、Disease Status** | |
| --- | --- |
| 1. **Date of generalized myasthenia gravis (gMG) diagnosis (YYYY / MM / DD):** | |
| 1. **What’s your class of Myasthenia Gravis Foundation of America (MGFA)?**   □ I □ II □ IV | |
| 1. **The Myasthenia Gravis Activities of Daily Living (MG-ADL) score** 2. **How have you been talking over the past week?**   □Normal □Intermittent slurring or nasal speech □Constant slurring, but can be understood □Difficult to understand speech   1. **How have you been chewing over the past week?**   □Normal □Fatigue with solid food □Fatigue with soft food □Gastric tube   1. **How have you been swallowing over the past week?**   □Normal □Rare episode of choking □Frequent choking necessitating changes in diet □Gastric tube   1. **How have you been breathing over the past week?**   □Normal □Shortness of breath with exertion □Shortness of breath at rest  □Ventilator dependence   1. **How have you been impairment of ability to brush teeth or comb hair over the past week?**   □ None □ Extra effort, but no rest periods needed □ Rest periods needed □ Cannot do one of these functions   1. **How have you been impairment of ability to arise from a chair over the past week?**   □ None □ Mild, sometimes uses arms □ Moderate, always uses arms □ Severe, requires assistance   1. **How have you been double vision over the past week?**   □ None □ Occurs, but not daily □ Daily, but not constant □ Constant   1. **How have you been eyelid droop over the past week?**   □ None □ Occurs, but not daily □ Daily, but not constant □ Constant | |
| 1. **What’s your result of serological testing?**   □ Negative □ Positive  □ Anti-AChR antibody positive  □ Anti-MuSK antibody positive  □ Anti-LRP4 antibody positive  □ Anti-transient receptor potential calcium channel antibody positive | |
| 1. **Do you have a thymectomy?**  □ Yes □ No | |
| 1. **Do you have any of the following comorbidities?**   □ Hyperthyroidism □ Hypothyroidism □ Psoriasis □ Eczema □ Urticaria □ Vitiligo □ Asthma □ Polymyositis □ Dermatomyositis □ Multiple sclerosis □ Neuromyelitis optica □Systemic lupus erythematosus □ Sjogren’s syndrome □ Vasculitis □ Scleroderma □ Systemic sclerosis □ Rheumatoid arthritis □ Hypertension □ Diabetes □ Hyperlipidemia □ Cancer □ Hepatitis B □ Tuberculosis □ Syphilis □ Psychiatric diseases □ Cerebral infarction □ Cerebral hemorrhage □ Leukopenia □ None □ Other: __________ | |
| **二、Treatment Status** | |
| 1. **Are you currently receiving treatment?**  □ Yes □ No | |
| 1. **When did you start treatment?** (YYYY / MM / DD) | |
| 1. **Have you received any of the following treatments?** (Multiple choices allowed) | |
| □ Pyridostigmine bromide | □ Prednisone acetate |
| □ Methylprednisolone  Administration route:  □ Oral  □ Intravenous | □ Azathioprine  Administration route: □ Oral |
| □ Tacrolimus | □ Mycophenolate mofetil |
| □ Cyclosporine | □ Methotrexate  Administration route:  □Oral  □ Intravenous  □ Subcutaneous |
| □ Cyclophosphamide  Administration route:  □ Oral  □ Intravenous  □ Subcutaneous | □ Rituximab |
| □ Eculizumab | □ Ravulizumab |
| □ Zilucoplan | □ Efgartigimod  Administration route: □ Intravenous  □ Subcutaneous |
| □ Intravenous immunoglobulin | □ Plasma exchange |
| □ Others  Administration route: □ Oral □ Intravenous □ Subcutaneous | |
| **Approximately how much do you pay out-of-pocket annually for myasthenia gravis (gMG) treatment?**  □ < ¥10,000 □ ¥10,000–30,000 □ ¥30,000–50,000 □ ¥50,000–100,000 □ > ¥100,000 | |
| **三、Patient Satisfaction** | |
| 1. **How satisfied are you with your current treatment?** **(1 = very dissatisfied, 10 = very satisfied. The level of satisfaction gradually increases from 1 to 10.)**   □ 1 □ 2 □ 3 □ 4 □ 5 □ 6 □ 7 □ 8 □ 9 □ 10 | |
| 1. **What side effects of your current treatment bother you the most?**   □ None □ Headache □ Infection (e.g., upper respiratory tract infection, meningococcal infection, urinary tract infection)  □ Gastrointestinal reactions (e.g., stomach ache, gasteremphraxis, diarrhea, nausea, vomiting, constipation with hematochezia)  □ Myelosuppression (e.g., anemia, leukopenia, thrombocytopenia)  □ Liver or kidney function impairment (e.g., abnormal liver function; abnormal kidney function)  □ Metabolic diseases (e.g., weight gain, central obesity, hypertension, hyperglycemia, menstrual irregularities)  □ Others _________________ | |
| 1. **What aspects of your current treatment are you most dissatisfied with?**   □ None □ Adverse drug reactions (side effects) □ Insufficient efficacy or no symptom improvement  □ High cost □ Inconvenient administration □ Too frequent dosing interval  □ Too infrequent dosing interval □ Other: _________________ | |

**Part Three: Discrete Choice Experiment on Treatment Preferences**

Instructions for Completion (Please read carefully before you start filling in!):

1. Please answer all questions carefully. Please fill in with a black rollerball pen, ballpoint pen, or fountain pen. If you make a mistake, draw a single line through the incorrect entry, correct it in the adjacent blank space, and sign with your initials along with the date of correction (e.g., "20.06 LZH 21.03.10". [Do not use correction fluid, erasers, or scribble over mistakes]).
2. This questionnaire requires you to make a choice between two different drugs (or treatment methods). We will describe the characteristics of the drugs or treatment methods from the following aspects:

**Onset time of action:**

The duration required for each treatment to improve the symptoms of generalized Myasthenia Gravis (gMG) from the start of medication.

- ≤ 2 weeks
- 2 weeks

**Administration route:**

- Intravenous infusion (infusion time ≥ 60 min)
- Subcutaneous injection (injection duration: 20 minutes)
- Oral

**Treatment frequency:**

- Once daily
- Weekly
- Biweekly
- Every-6-month
- Daily 2 to 3 times

**Risk of metabolic diseases:**

Metabolic diseases include weight gain, central obesity, hypertension, hyperglycemia. The occurrence risk included: low, moderate, and high level.

**Risk of infection:**

Infections include upper respiratory tract infections, meningococcal infections, and urinary tract infections. The occurrence risk included: low, moderate, and high level.

**Risk of myelosuppression:**

Myelosuppression includes anemia, leukopenia, and thrombocytopenia. The occurrence risk included: low, moderate, and high level.

**Risk of liver and kidney function impairment:**

Liver and kidney function impairment includes abnormal liver and kidney function. The occurrence risk included: low, moderate, and high level.

**Out-of-pocket annual cost:**

For all costs associated with the treatment, if you have medical insurance, they refer to the costs after medical insurance reimbursement.

- ¥10,000
- ¥30,000
- ¥50,000
- ¥100,000

We would like to understand how you make choices between different drugs or treatment methods. For each question, you need to consider the following:

- If you can only choose one of the two treatments (Drug A and Drug B), which one will you select?
- If the treatment you have chosen actually exists in reality, are you willing to use it?

Below is an example, followed by 10 questions that require your answers. Please read the example before completing the questionnaire.

***Example***

| Which option will you choose? (**Select only one option. Mark your choice with a “√”, likeR）** | | |
| --- | --- | --- |
| **Attribute/Level** | **Drug A** | **Drug B** |
| Onset time of action | ≤ 2 weeks | > 2 weeks |
| Administration route | Oral | Intravenous infusion (infusion time ≥ 60 min) |
| Treatment frequency | Once daily | Once weekly |
| Risk of metabolic diseases (e.g., weight gain, central obesity, hypertension, hyperglycemia) | Low | Moderate |
| Risk of infection (e.g., upper respiratory tract infection, meningococcal infection, urinary tract infection) | Moderate | High |
| Risk of myelosuppression (e.g., anemia, leukopenia, thrombocytopenia) | Low | Moderate |
| Risk of liver and kidney function impairment (e.g., abnormal liver function; abnormal kidney function) | High | Low |
| Out-of-pocket annual cost | ¥30,000 | ¥50,000 |
| Which do you prefer? | **□** | **□** |
| In reality, would you be willing to choose the treatment option you selected above? | **□**Yes  **□**No | |

*The above example demonstrates that if you choose Drug A, it indicates a preference for a medication with the following characteristics compared to Drug B: onset time of action within ≤ 2 weeks, oral administration, once daily dosing, low risk of metabolic diseases, moderate risk of infection, low risk of myelosuppression, high risk of liver and kidney function impairment, and an annual out-of-pocket cost of ¥30,000. Additionally, you would be willing to use Drug A if it were available in reality.*

After you have fully understood the above explanations, please proceed to the official preference questionnaire and make your selections for the following questions.

**Formal question (Versions A)**

| **Question 1: Which option will you choose? (Select only one option. Mark your choice with a “√”, likeR）** | | |
| --- | --- | --- |
| **Attribute/Level** | **Drug A** | **Drug B** |
| Onset time of action | > 2 weeks | ≤ 2 weeks |
| Administration route | Subcutaneous injection (injection duration: 20 minutes) | Intravenous infusion (infusion time ≥ 60 min) |
| Treatment frequency | Once daily | Biweekly |
| Risk of metabolic diseases (e.g., weight gain, central obesity, hypertension, hyperglycemia, menstrual irregularities) | Moderate | Low |
| Risk of infection (e.g., upper respiratory tract infection, meningococcal infection, urinary tract infection) | Moderate | Low |
| Risk of myelosuppression (e.g., anemia, leukopenia, thrombocytopenia) | High | Low |
| Risk of liver and kidney function impairment (e.g., abnormal liver function; abnormal kidney function) | Moderate | Low |
| Out-of-pocket annual cost | ¥100,000 | ¥30,000 |
| Which do you prefer? | **□** | **□** |
| In reality, would you be willing to choose the treatment option you selected above? | **□**Yes  **□**No | |

| **Question 2: Which option will you choose? (Select only one option. Mark your choice with a “√”, likeR）** | | |
| --- | --- | --- |
| **Attribute/Level** | **Drug A** | **Drug B** |
| Onset time of action | ≤ 2 weeks | > 2 weeks |
| Administration route | Subcutaneous injection (injection duration: 20 minutes) | Intravenous infusion (infusion time ≥ 60 min) |
| Treatment frequency | Once daily | Daily 2 to 3 times |
| Risk of metabolic diseases (e.g., weight gain, central obesity, hypertension, hyperglycemia, menstrual irregularities) | Low | High |
| Risk of infection (e.g., upper respiratory tract infection, meningococcal infection, urinary tract infection) | High | Low |
| Risk of myelosuppression (e.g., anemia, leukopenia, thrombocytopenia) | Low | High |
| Risk of liver and kidney function impairment (e.g., abnormal liver function; abnormal kidney function) | High | Low |
| Out-of-pocket annual cost | ¥50,000 | ¥100,000 |
| Which do you prefer? | **□** | **□** |
| In reality, would you be willing to choose the treatment option you selected above? | **□**Yes  **□**No | |

| **Question 3: Which option will you choose? (Select only one option. Mark your choice with a “√”, likeR）** | | |
| --- | --- | --- |
| **Attribute/Level** | **Drug A** | **Drug B** |
| Onset time of action | > 2 weeks | ≤ 2 weeks |
| Administration route | Subcutaneous injection (injection duration: 20 minutes) | Oral |
| Treatment frequency | Every-6-month | Weekly |
| Risk of metabolic diseases (e.g., weight gain, central obesity, hypertension, hyperglycemia, menstrual irregularities) | Moderate | High |
| Risk of infection (e.g., upper respiratory tract infection, meningococcal infection, urinary tract infection) | Moderate | High |
| Risk of myelosuppression (e.g., anemia, leukopenia, thrombocytopenia) | High | Moderate |
| Risk of liver and kidney function impairment (e.g., abnormal liver function; abnormal kidney function) | Low | High |
| Out-of-pocket annual cost | ¥50,000 | ¥100,000 |
| Which do you prefer? | **□** | **□** |
| In reality, would you be willing to choose the treatment option you selected above? | **□**Yes  **□**No | |

| **Question 4: Which option will you choose? (Select only one option. Mark your choice with a “√”, likeR）** | | |
| --- | --- | --- |
| **Attribute/Level** | **Drug A** | **Drug B** |
| Onset time of action | > 2 weeks | ≤ 2 weeks |
| Administration route | Intravenous infusion (infusion time ≥ 60 min) | Oral |
| Treatment frequency | Once daily | Every-6-month |
| Risk of metabolic diseases (e.g., weight gain, central obesity, hypertension, hyperglycemia, menstrual irregularities) | Moderate | High |
| Risk of infection (e.g., upper respiratory tract infection, meningococcal infection, urinary tract infection) | Low | Moderate |
| Risk of myelosuppression (e.g., anemia, leukopenia, thrombocytopenia) | Moderate | High |
| Risk of liver and kidney function impairment (e.g., abnormal liver function; abnormal kidney function) | Moderate | High |
| Out-of-pocket annual cost | ¥100,000 | ¥10,000 |
| Which do you prefer? | **□** | **□** |
| In reality, would you be willing to choose the treatment option you selected above? | **□**Yes  **□**No | |

| **Question 5: Which option will you choose? (Select only one option. Mark your choice with a “√”, likeR）** | | |
| --- | --- | --- |
| **Attribute/Level** | **Drug A** | **Drug B** |
| Onset time of action | > 2 weeks | ≤ 2 weeks |
| Administration route | Oral | Intravenous infusion (infusion time ≥ 60 min) |
| Treatment frequency | Once daily | Daily 2 to 3 times |
| Risk of metabolic diseases (e.g., weight gain, central obesity, hypertension, hyperglycemia, menstrual irregularities) | High | Moderate |
| Risk of infection (e.g., upper respiratory tract infection, meningococcal infection, urinary tract infection) | Low | Moderate |
| Risk of myelosuppression (e.g., anemia, leukopenia, thrombocytopenia) | Moderate | Low |
| Risk of liver and kidney function impairment (e.g., abnormal liver function; abnormal kidney function) | Low | Moderate |
| Out-of-pocket annual cost | ¥30,000 | ¥100,000 |
| Which do you prefer? | **□** | **□** |
| In reality, would you be willing to choose the treatment option you selected above? | **□**Yes  **□**No | |

| **Question 6: Which option will you choose? (Select only one option. Mark your choice with a “√”, likeR）** | | |
| --- | --- | --- |
| **Attribute/Level** | **Drug A** | **Drug B** |
| Onset time of action | > 2 weeks | ≤ 2 weeks |
| Administration route | Subcutaneous injection (injection duration: 20 minutes) | Intravenous infusion (infusion time ≥ 60 min) |
| Treatment frequency | Daily 2 to 3 times | Every-6-month |
| Risk of metabolic diseases (e.g., weight gain, central obesity, hypertension, hyperglycemia, menstrual irregularities) | Low | Moderate |
| Risk of infection (e.g., upper respiratory tract infection, meningococcal infection, urinary tract infection) | Moderate | High |
| Risk of myelosuppression (e.g., anemia, leukopenia, thrombocytopenia) | Low | High |
| Risk of liver and kidney function impairment (e.g., abnormal liver function; abnormal kidney function) | Moderate | High |
| Out-of-pocket annual cost | ¥50,000 | ¥10,000 |
| Which do you prefer? | **□** | **□** |
| In reality, would you be willing to choose the treatment option you selected above? | **□**Yes  **□**No | |

| **Question 7: Which option will you choose? (Select only one option. Mark your choice with a “√”, likeR）** | | |
| --- | --- | --- |
| **Attribute/Level** | **Drug A** | **Drug B** |
| Onset time of action | > 2 weeks | ≤ 2 weeks |
| Administration route | Subcutaneous injection (injection duration: 20 minutes) | Intravenous infusion (infusion time ≥ 60 min) |
| Treatment frequency | Daily 2 to 3 times | Weekly |
| Risk of metabolic diseases (e.g., weight gain, central obesity, hypertension, hyperglycemia, menstrual irregularities) | High | Low |
| Risk of infection (e.g., upper respiratory tract infection, meningococcal infection, urinary tract infection) | High | Moderate |
| Risk of myelosuppression (e.g., anemia, leukopenia, thrombocytopenia) | Low | High |
| Risk of liver and kidney function impairment (e.g., abnormal liver function; abnormal kidney function) | High | Low |
| Out-of-pocket annual cost | ¥10,000 | ¥50,000 |
| Which do you prefer? | **□** | **□** |
| In reality, would you be willing to choose the treatment option you selected above? | **□**Yes  **□**No | |

| **Question 8: Which option will you choose? (Select only one option. Mark your choice with a “√”, likeR）** | | |
| --- | --- | --- |
| **Attribute/Level** | **Drug A** | **Drug B** |
| Onset time of action | ≤ 2 weeks | > 2 weeks |
| Administration route | Intravenous infusion (infusion time ≥ 60 min) | Subcutaneous injection (injection duration: 20 minutes) |
| Treatment frequency | Biweekly | Every-6-month |
| Risk of metabolic diseases (e.g., weight gain, central obesity, hypertension, hyperglycemia, menstrual irregularities) | Moderate | Low |
| Risk of infection (e.g., upper respiratory tract infection, meningococcal infection, urinary tract infection) | High | Low |
| Risk of myelosuppression (e.g., anemia, leukopenia, thrombocytopenia) | Low | Moderate |
| Risk of liver and kidney function impairment (e.g., abnormal liver function; abnormal kidney function) | Moderate | High |
| Out-of-pocket annual cost | ¥30,000 | ¥100,000 |
| Which do you prefer? | **□** | **□** |
| In reality, would you be willing to choose the treatment option you selected above? | **□**Yes  **□**No | |

| **Question 9: Which option will you choose? (Select only one option. Mark your choice with a “√”, likeR）** | | |
| --- | --- | --- |
| **Attribute/Level** | **Drug A** | **Drug B** |
| Onset time of action | > 2 weeks | ≤ 2 weeks |
| Administration route | Intravenous infusion (infusion time ≥ 60 min) | Oral |
| Treatment frequency | Once daily | Every-6-month |
| Risk of metabolic diseases (e.g., weight gain, central obesity, hypertension, hyperglycemia, menstrual irregularities) | Moderate | High |
| Risk of infection (e.g., upper respiratory tract infection, meningococcal infection, urinary tract infection) | Low | Moderate |
| Risk of myelosuppression (e.g., anemia, leukopenia, thrombocytopenia) | Moderate | High |
| Risk of liver and kidney function impairment (e.g., abnormal liver function; abnormal kidney function) | Moderate | High |
| Out-of-pocket annual cost | ¥100,000 | ¥10,000 |
| Which do you prefer? | **□** | **□** |
| In reality, would you be willing to choose the treatment option you selected above? | **□**Yes  **□**No | |

**Question 10: Please indicate your level of agreement or disagreement with each of the following statements based on the information and questions in this questionnaire. (Select only one option and mark it with a "√".)**

|  | **Strongly disagree** | **Disagree** | **Uncertain** | **Agree** | **Strongly agree** |
| --- | --- | --- | --- | --- | --- |
| 1. I understand the idea of making choices between different treatments | **□** | **□** | **□** | **□** | **□** |
| 1. When choosing treatments, I need more information than what is provided here. | **□** | **□** | **□** | **□** | **□** |
| 1. I believe my choices will influence which treatments are offered in the future. | **□** | **□** | **□** | **□** | **□** |
| 1. The available treatment options seem reasonable to me. | **□** | **□** | **□** | **□** | **□** |
| 1. The more questions I answer, the easier it is to make a decision. | **□** | **□** | **□** | **□** | **□** |
| 1. I find it confusing to choose between different treatment methods. | **□** | **□** | **□** | **□** | **□** |

**Formal question (Versions B)**

| **Question 1: Which option will you choose? (Select only one option. Mark your choice with a “√”, likeR）** | | |
| --- | --- | --- |
| **Attribute/Level** | **Drug A** | **Drug B** |
| Onset time of action | > 2 weeks | ≤ 2 weeks |
| Administration route | Intravenous infusion (infusion time ≥ 60 min) | Oral |
| Treatment frequency | Daily 2 to 3 times | Weekly |
| Risk of metabolic diseases (e.g., weight gain, central obesity, hypertension, hyperglycemia, menstrual irregularities) | Low | Moderate |
| Risk of infection (e.g., upper respiratory tract infection, meningococcal infection, urinary tract infection) | High | Low |
| Risk of myelosuppression (e.g., anemia, leukopenia, thrombocytopenia) | Moderate | Low |
| Risk of liver and kidney function impairment (e.g., abnormal liver function; abnormal kidney function) | High | Moderate |
| Out-of-pocket annual cost | ¥30,000 | ¥100,000 |
| Which do you prefer? | **□** | **□** |
| In reality, would you be willing to choose the treatment option you selected above? | **□**Yes  **□**No | |

| **Question 2: Which option will you choose? (Select only one option. Mark your choice with a “√”, likeR）** | | |
| --- | --- | --- |
| **Attribute/Level** | **Drug A** | **Drug B** |
| Onset time of action | > 2 weeks | ≤ 2 weeks |
| Administration route | Intravenous infusion (infusion time ≥ 60 min) | Subcutaneous injection (injection duration: 20 minutes) |
| Treatment frequency | Daily 2 to 3 times | Biweekly |
| Risk of metabolic diseases (e.g., weight gain, central obesity, hypertension, hyperglycemia, menstrual irregularities) | High | Low |
| Risk of infection (e.g., upper respiratory tract infection, meningococcal infection, urinary tract infection) | High | Low |
| Risk of myelosuppression (e.g., anemia, leukopenia, thrombocytopenia) | Low | High |
| Risk of liver and kidney function impairment (e.g., abnormal liver function; abnormal kidney function) | Low | Moderate |
| Out-of-pocket annual cost | ¥50,000 | ¥10,000 |
| Which do you prefer? | **□** | **□** |
| In reality, would you be willing to choose the treatment option you selected above? | **□**Yes  **□**No | |

| **Question 3: Which option will you choose? (Select only one option. Mark your choice with a “√”, likeR）** | | |
| --- | --- | --- |
| **Attribute/Level** | **Drug A** | **Drug B** |
| Onset time of action | ≤ 2 weeks | > 2 weeks |
| Administration route | Oral | Subcutaneous injection (injection duration: 20 minutes) |
| Treatment frequency | Once daily | Weekly |
| Risk of metabolic diseases (e.g., weight gain, central obesity, hypertension, hyperglycemia, menstrual irregularities) | High | Low |
| Risk of infection (e.g., upper respiratory tract infection, meningococcal infection, urinary tract infection) | Low | High |
| Risk of myelosuppression (e.g., anemia, leukopenia, thrombocytopenia) | Low | Moderate |
| Risk of liver and kidney function impairment (e.g., abnormal liver function; abnormal kidney function) | High | Low |
| Out-of-pocket annual cost | ¥50,000 | ¥10,000 |
| Which do you prefer? | **□** | **□** |
| In reality, would you be willing to choose the treatment option you selected above? | **□**Yes  **□**No | |

| **Question 4: Which option will you choose? (Select only one option. Mark your choice with a “√”, likeR）** | | |
| --- | --- | --- |
| **Attribute/Level** | **Drug A** | **Drug B** |
| Onset time of action | > 2 weeks | ≤ 2 weeks |
| Administration route | Oral | Subcutaneous injection (injection duration: 20 minutes) |
| Treatment frequency | Once daily | Weekly |
| Risk of metabolic diseases (e.g., weight gain, central obesity, hypertension, hyperglycemia, menstrual irregularities) | Low | High |
| Risk of infection (e.g., upper respiratory tract infection, meningococcal infection, urinary tract infection) | High | Low |
| Risk of myelosuppression (e.g., anemia, leukopenia, thrombocytopenia) | High | Moderate |
| Risk of liver and kidney function impairment (e.g., abnormal liver function; abnormal kidney function) | Moderate | High |
| Out-of-pocket annual cost | ¥10,000 | ¥50,000 |
| Which do you prefer? | **□** | **□** |
| In reality, would you be willing to choose the treatment option you selected above? | **□**Yes  **□**No | |

| **Question 5: Which option will you choose? (Select only one option. Mark your choice with a “√”, likeR）** | | |
| --- | --- | --- |
| **Attribute/Level** | **Drug A** | **Drug B** |
| Onset time of action | > 2 weeks | ≤ 2 weeks |
| Administration route | Subcutaneous injection (injection duration: 20 minutes) | Intravenous infusion (infusion time ≥ 60 min) |
| Treatment frequency | Weekly | Every-6-month |
| Risk of metabolic diseases (e.g., weight gain, central obesity, hypertension, hyperglycemia, menstrual irregularities) | High | Low |
| Risk of infection (e.g., upper respiratory tract infection, meningococcal infection, urinary tract infection) | Moderate | Low |
| Risk of myelosuppression (e.g., anemia, leukopenia, thrombocytopenia) | Low | Moderate |
| Risk of liver and kidney function impairment (e.g., abnormal liver function; abnormal kidney function) | Low | Moderate |
| Out-of-pocket annual cost | ¥30,000 | ¥50,000 |
| Which do you prefer? | **□** | **□** |
| In reality, would you be willing to choose the treatment option you selected above? | **□**Yes  **□**No | |

| **Question 6: Which option will you choose? (Select only one option. Mark your choice with a “√”, likeR）** | | |
| --- | --- | --- |
| **Attribute/Level** | **Drug A** | **Drug B** |
| Onset time of action | ≤ 2 weeks | > 2 weeks |
| Administration route | Intravenous infusion (infusion time ≥ 60 min) | Oral |
| Treatment frequency | Once daily | Biweekly |
| Risk of metabolic diseases (e.g., weight gain, central obesity, hypertension, hyperglycemia, menstrual irregularities) | High | Moderate |
| Risk of infection (e.g., upper respiratory tract infection, meningococcal infection, urinary tract infection) | Moderate | Low |
| Risk of myelosuppression (e.g., anemia, leukopenia, thrombocytopenia) | Moderate | High |
| Risk of liver and kidney function impairment (e.g., abnormal liver function; abnormal kidney function) | Moderate | High |
| Out-of-pocket annual cost | ¥10,000 | ¥30,000 |
| Which do you prefer? | **□** | **□** |
| In reality, would you be willing to choose the treatment option you selected above? | **□**Yes  **□**No | |

| **Question 7: Which option will you choose? (Select only one option. Mark your choice with a “√”, likeR）** | | |
| --- | --- | --- |
| **Attribute/Level** | **Drug A** | **Drug B** |
| Onset time of action | ≤ 2 weeks | > 2 weeks |
| Administration route | Intravenous infusion (infusion time ≥ 60 min) | Oral |
| Treatment frequency | Once daily | Biweekly |
| Risk of metabolic diseases (e.g., weight gain, central obesity, hypertension, hyperglycemia, menstrual irregularities) | Low | Moderate |
| Risk of infection (e.g., upper respiratory tract infection, meningococcal infection, urinary tract infection) | Low | High |
| Risk of myelosuppression (e.g., anemia, leukopenia, thrombocytopenia) | Low | Moderate |
| Risk of liver and kidney function impairment (e.g., abnormal liver function; abnormal kidney function) | Low | Moderate |
| Out-of-pocket annual cost | ¥30,000 | ¥50,000 |
| Which do you prefer? | **□** | **□** |
| In reality, would you be willing to choose the treatment option you selected above? | **□**Yes  **□**No | |

| **Question 8: Which option will you choose? (Select only one option. Mark your choice with a “√”, likeR）** | | |
| --- | --- | --- |
| **Attribute/Level** | **Drug A** | **Drug B** |
| Onset time of action | > 2 weeks | ≤ 2 weeks |
| Administration route | Oral | Subcutaneous injection (injection duration: 20 minutes) |
| Treatment frequency | Weekly | Biweekly |
| Risk of metabolic diseases (e.g., weight gain, central obesity, hypertension, hyperglycemia, menstrual irregularities) | High | Moderate |
| Risk of infection (e.g., upper respiratory tract infection, meningococcal infection, urinary tract infection) | High | Moderate |
| Risk of myelosuppression (e.g., anemia, leukopenia, thrombocytopenia) | High | Moderate |
| Risk of liver and kidney function impairment (e.g., abnormal liver function; abnormal kidney function) | Moderate | High |
| Out-of-pocket annual cost | ¥50,000 | ¥100,000 |
| Which do you prefer? | **□** | **□** |
| In reality, would you be willing to choose the treatment option you selected above? | **□**Yes  **□**No | |

| **Question 9: Which option will you choose? (Select only one option. Mark your choice with a “√”, likeR）** | | |
| --- | --- | --- |
| **Attribute/Level** | **Drug A** | **Drug B** |
| Onset time of action | > 2 weeks | ≤ 2 weeks |
| Administration route | Oral | Subcutaneous injection (injection duration: 20 minutes) |
| Treatment frequency | Once daily | Weekly |
| Risk of metabolic diseases (e.g., weight gain, central obesity, hypertension, hyperglycemia, menstrual irregularities) | Low | High |
| Risk of infection (e.g., upper respiratory tract infection, meningococcal infection, urinary tract infection) | High | Low |
| Risk of myelosuppression (e.g., anemia, leukopenia, thrombocytopenia) | High | Moderate |
| Risk of liver and kidney function impairment (e.g., abnormal liver function; abnormal kidney function) | Moderate | High |
| Out-of-pocket annual cost | ¥10,000 | ¥50,000 |
| Which do you prefer? | **□** | **□** |
| In reality, would you be willing to choose the treatment option you selected above? | **□**Yes  **□**No | |

**Question 10: Please indicate your level of agreement or disagreement with each of the following statements based on the information and questions in this questionnaire. (Select only one option and mark it with a "√".)**

|  | **Strongly disagree** | **Disagree** | **Uncertain** | **Agree** | **Strongly agree** |
| --- | --- | --- | --- | --- | --- |
| 1. I understand the idea of making choices between different treatments | **□** | **□** | **□** | **□** | **□** |
| 1. When choosing treatments, I need more information than what is provided here. | **□** | **□** | **□** | **□** | **□** |
| 1. I believe my choices will influence which treatments are offered in the future. | **□** | **□** | **□** | **□** | **□** |
| 1. The available treatment options seem reasonable to me. | **□** | **□** | **□** | **□** | **□** |
| 1. The more questions I answer, the easier it is to make a decision. | **□** | **□** | **□** | **□** | **□** |
| 1. I find it confusing to choose between different treatment methods. | **□** | **□** | **□** | **□** | **□** |

**Formal question (Versions C)**

| **Question 1: Which option will you choose? (Select only one option. Mark your choice with a “√”, likeR）** | | |
| --- | --- | --- |
| **Attribute/Level** | **Drug A** | **Drug B** |
| Onset time of action | ≤ 2 weeks | > 2 weeks |
| Administration route | Subcutaneous injection (injection duration: 20 minutes) | Intravenous infusion (infusion time ≥ 60 min) |
| Treatment frequency | Every-6-month | Biweekly |
| Risk of metabolic diseases (e.g., weight gain, central obesity, hypertension, hyperglycemia, menstrual irregularities) | Moderate | High |
| Risk of infection (e.g., upper respiratory tract infection, meningococcal infection, urinary tract infection) | High | Moderate |
| Risk of myelosuppression (e.g., anemia, leukopenia, thrombocytopenia) | Moderate | High |
| Risk of liver and kidney function impairment (e.g., abnormal liver function; abnormal kidney function) | Moderate | High |
| Out-of-pocket annual cost | ¥30,000 | ¥10,000 |
| Which do you prefer? | **□** | **□** |
| In reality, would you be willing to choose the treatment option you selected above? | **□**Yes  **□**No | |

| **Question 2: Which option will you choose? (Select only one option. Mark your choice with a “√”, likeR）** | | |
| --- | --- | --- |
| **Attribute/Level** | **Drug A** | **Drug B** |
| Onset time of action | > 2 weeks | ≤ 2 weeks |
| Administration route | Oral | Subcutaneous injection (injection duration: 20 minutes) |
| Treatment frequency | Weekly | Every-6-month |
| Risk of metabolic diseases (e.g., weight gain, central obesity, hypertension, hyperglycemia, menstrual irregularities) | Moderate | High |
| Risk of infection (e.g., upper respiratory tract infection, meningococcal infection, urinary tract infection) | Moderate | High |
| Risk of myelosuppression (e.g., anemia, leukopenia, thrombocytopenia) | Moderate | High |
| Risk of liver and kidney function impairment (e.g., abnormal liver function; abnormal kidney function) | High | Low |
| Out-of-pocket annual cost | ¥10,000 | ¥100,000 |
| Which do you prefer? | **□** | **□** |
| In reality, would you be willing to choose the treatment option you selected above? | **□**Yes  **□**No | |

| **Question 3: Which option will you choose? (Select only one option. Mark your choice with a “√”, likeR）** | | |
| --- | --- | --- |
| **Attribute/Level** | **Drug A** | **Drug B** |
| Onset time of action | ≤ 2 weeks | > 2 weeks |
| Administration route | Oral | Intravenous infusion (infusion time ≥ 60 min) |
| Treatment frequency | Biweekly | Weekly |
| Risk of metabolic diseases (e.g., weight gain, central obesity, hypertension, hyperglycemia, menstrual irregularities) | High | Moderate |
| Risk of infection (e.g., upper respiratory tract infection, meningococcal infection, urinary tract infection) | Moderate | Low |
| Risk of myelosuppression (e.g., anemia, leukopenia, thrombocytopenia) | Moderate | Low |
| Risk of liver and kidney function impairment (e.g., abnormal liver function; abnormal kidney function) | Low | High |
| Out-of-pocket annual cost | ¥100,000 | ¥10,000 |
| Which do you prefer? | **□** | **□** |
| In reality, would you be willing to choose the treatment option you selected above? | **□**Yes  **□**No | |

| **Question 4: Which option will you choose? (Select only one option. Mark your choice with a “√”, likeR）** | | |
| --- | --- | --- |
| **Attribute/Level** | **Drug A** | **Drug B** |
| Onset time of action | ≤ 2 weeks | > 2 weeks |
| Administration route | Subcutaneous injection (injection duration: 20 minutes) | Oral |
| Treatment frequency | Daily 2 to 3 times | Every-6-month |
| Risk of metabolic diseases (e.g., weight gain, central obesity, hypertension, hyperglycemia, menstrual irregularities) | High | Moderate |
| Risk of infection (e.g., upper respiratory tract infection, meningococcal infection, urinary tract infection) | Low | High |
| Risk of myelosuppression (e.g., anemia, leukopenia, thrombocytopenia) | High | Low |
| Risk of liver and kidney function impairment (e.g., abnormal liver function; abnormal kidney function) | Moderate | Low |
| Out-of-pocket annual cost | ¥30,000 | ¥100,000 |
| Which do you prefer? | **□** | **□** |
| In reality, would you be willing to choose the treatment option you selected above? | **□**Yes  **□**No | |

| **Question 5: Which option will you choose? (Select only one option. Mark your choice with a “√”, likeR）** | | |
| --- | --- | --- |
| **Attribute/Level** | **Drug A** | **Drug B** |
| Onset time of action | ≤ 2 weeks | > 2 weeks |
| Administration route | Oral | Subcutaneous injection (injection duration: 20 minutes) |
| Treatment frequency | Daily 2 to 3 times | Biweekly |
| Risk of metabolic diseases (e.g., weight gain, central obesity, hypertension, hyperglycemia, menstrual irregularities) | Moderate | High |
| Risk of infection (e.g., upper respiratory tract infection, meningococcal infection, urinary tract infection) | Moderate | Low |
| Risk of myelosuppression (e.g., anemia, leukopenia, thrombocytopenia) | High | Low |
| Risk of liver and kidney function impairment (e.g., abnormal liver function; abnormal kidney function) | High | Moderate |
| Out-of-pocket annual cost | ¥30,000 | ¥10,000 |
| Which do you prefer? | **□** | **□** |
| In reality, would you be willing to choose the treatment option you selected above? | **□**Yes  **□**No | |

| **Question 6: Which option will you choose? (Select only one option. Mark your choice with a “√”, likeR）** | | |
| --- | --- | --- |
| **Attribute/Level** | **Drug A** | **Drug B** |
| Onset time of action | ≤ 2 weeks | > 2 weeks |
| Administration route | Oral | Intravenous infusion (infusion time ≥ 60 min) |
| Treatment frequency | Daily 2 to 3 times | Weekly |
| Risk of metabolic diseases (e.g., weight gain, central obesity, hypertension, hyperglycemia, menstrual irregularities) | Moderate | Low |
| Risk of infection (e.g., upper respiratory tract infection, meningococcal infection, urinary tract infection) | Low | High |
| Risk of myelosuppression (e.g., anemia, leukopenia, thrombocytopenia) | Moderate | High |
| Risk of liver and kidney function impairment (e.g., abnormal liver function; abnormal kidney function) | Low | High |
| Out-of-pocket annual cost | ¥10,000 | ¥100,000 |
| Which do you prefer? | **□** | **□** |
| In reality, would you be willing to choose the treatment option you selected above? | **□**Yes  **□**No | |

| **Question 7: Which option will you choose? (Select only one option. Mark your choice with a “√”, likeR）** | | |
| --- | --- | --- |
| **Attribute/Level** | **Drug A** | **Drug B** |
| Onset time of action | ≤ 2 weeks | > 2 weeks |
| Administration route | Subcutaneous injection (injection duration: 20 minutes) | Oral |
| Treatment frequency | Once daily | Every-6-month |
| Risk of metabolic diseases (e.g., weight gain, central obesity, hypertension, hyperglycemia, menstrual irregularities) | Moderate | Low |
| Risk of infection (e.g., upper respiratory tract infection, meningococcal infection, urinary tract infection) | High | Moderate |
| Risk of myelosuppression (e.g., anemia, leukopenia, thrombocytopenia) | High | Low |
| Risk of liver and kidney function impairment (e.g., abnormal liver function; abnormal kidney function) | Low | Moderate |
| Out-of-pocket annual cost | ¥50,000 | ¥30,000 |
| Which do you prefer? | **□** | **□** |
| In reality, would you be willing to choose the treatment option you selected above? | **□**Yes  **□**No | |

| **Question 8: Which option will you choose? (Select only one option. Mark your choice with a “√”, likeR）** | | |
| --- | --- | --- |
| **Attribute/Level** | **Drug A** | **Drug B** |
| Onset time of action | > 2 weeks | ≤ 2 weeks |
| Administration route | Intravenous infusion (infusion time ≥ 60 min) | Oral |
| Treatment frequency | Every-6-month | Daily 2 to 3 times |
| Risk of metabolic diseases (e.g., weight gain, central obesity, hypertension, hyperglycemia, menstrual irregularities) | High | Low |
| Risk of infection (e.g., upper respiratory tract infection, meningococcal infection, urinary tract infection) | Moderate | High |
| Risk of myelosuppression (e.g., anemia, leukopenia, thrombocytopenia) | Moderate | Low |
| Risk of liver and kidney function impairment (e.g., abnormal liver function; abnormal kidney function) | Moderate | Low |
| Out-of-pocket annual cost | ¥30,000 | ¥10,000 |
| Which do you prefer? | **□** | **□** |
| In reality, would you be willing to choose the treatment option you selected above? | **□**Yes  **□**No | |

| **Question 9: Which option will you choose? (Select only one option. Mark your choice with a “√”, likeR）** | | |
| --- | --- | --- |
| **Attribute/Level** | **Drug A** | **Drug B** |
| Onset time of action | ≤ 2 weeks | > 2 weeks |
| Administration route | Subcutaneous injection (injection duration: 20 minutes) | Oral |
| Treatment frequency | Daily 2 to 3 times | Every-6-month |
| Risk of metabolic diseases (e.g., weight gain, central obesity, hypertension, hyperglycemia, menstrual irregularities) | High | Moderate |
| Risk of infection (e.g., upper respiratory tract infection, meningococcal infection, urinary tract infection) | Low | High |
| Risk of myelosuppression (e.g., anemia, leukopenia, thrombocytopenia) | High | Low |
| Risk of liver and kidney function impairment (e.g., abnormal liver function; abnormal kidney function) | Moderate | Low |
| Out-of-pocket annual cost | ¥30,000 | ¥100,000 |
| Which do you prefer? | **□** | **□** |
| In reality, would you be willing to choose the treatment option you selected above? | **□**Yes  **□**No | |

**Question 10: Please indicate your level of agreement or disagreement with each of the following statements based on the information and questions in this questionnaire. (Select only one option and mark it with a "√".)**

|  | **Strongly disagree** | **Disagree** | **Uncertain** | **Agree** | **Strongly agree** |
| --- | --- | --- | --- | --- | --- |
| 1. I understand the idea of making choices between different treatments | **□** | **□** | **□** | **□** | **□** |
| 1. When choosing treatments, I need more information than what is provided here. | **□** | **□** | **□** | **□** | **□** |
| 1. I believe my choices will influence which treatments are offered in the future. | **□** | **□** | **□** | **□** | **□** |
| 1. The available treatment options seem reasonable to me. | **□** | **□** | **□** | **□** | **□** |
| 1. The more questions I answer, the easier it is to make a decision. | **□** | **□** | **□** | **□** | **□** |
| 1. I find it confusing to choose between different treatment methods. | **□** | **□** | **□** | **□** | **□** |

**Questionnaire Confirmation Statement**

I, as the investigator, confirm that the recorded questionnaire data is true, accurate, and reliable.

Name of investigator: ______________________

Date (YYYY/MM/DD): ______________________
